# Plasma metabolites associated with colorectal cancer stage: Findings from an international consortium

**DOI:** 10.1002/ijc.32666

**Published:** 2019-10-10

**Authors:** Anne J.M.R. Geijsen, Eline H. van Roekel, Fränzel J.B. van Duijnhoven, David Achaintre, Thomas Bachleitner‐Hofmann, Andreas Baierl, Michael M. Bergmann, Jürgen Boehm, Martijn J.L. Bours, Hermann Brenner, Stéphanie O. Breukink, Stefanie Brezina, Jenny Chang‐Claude, Esther Herpel, Johannes H.W. de Wilt, Audrey Gicquiau, Biljana Gigic, Tanja Gumpenberger, Bibi M.E. Hansson, Michael Hoffmeister, Andreana N. Holowatyj, Judith Karner‐Hanusch, Pekka Keski‐Rahkonen, Eric T.P. Keulen, Janna L. Koole, Gernot Leeb, Jennifer Ose, Peter Schirmacher, Martin A. Schneider, Petra Schrotz‐King, Anton Stift, Arve Ulvik, F. Jeroen Vogelaar, Evertine Wesselink, Moniek van Zutphen, Andrea Gsur, Nina Habermann, Ellen Kampman, Augustin Scalbert, Per M. Ueland, Alexis B. Ulrich, Cornelia M. Ulrich, Matty P. Weijenberg, Dieuwertje E. Kok

**Affiliations:** ^1^ Division of Human Nutrition and Health Wageningen University & Research Wageningen The Netherlands; ^2^ Department of Epidemiology, GROW School for Oncology and Developmental Biology Maastricht University Maastricht The Netherlands; ^3^ Biomarkers Group International Agency for Research on Cancer Lyon France; ^4^ Department of Surgery Medical University Vienna Vienna Austria; ^5^ Department of Statistics and Operations Research University of Vienna Vienna Austria; ^6^ Huntsman Cancer Institute Salt Lake City UT; ^7^ Department of Population Health Sciences University of Utah Salt Lake City UT; ^8^ Division of Preventive Oncology National Center for Tumor Diseases and German Cancer Research Center Heidelberg Germany; ^9^ Division of Clinical Epidemiology and Aging Research German Cancer Research Center (DKFZ) Heidelberg Germany; ^10^ German Cancer Consortium (DKTK) German Cancer Research Center (DKFZ) Heidelberg Germany; ^11^ Department of Surgery, GROW School for Oncology and Development Biology Maastricht University Maastricht The Netherlands; ^12^ Institute of Cancer Research, Department of Medicine I Medical University of Vienna Vienna Austria; ^13^ Division of Cancer Epidemiology German Cancer Research Center Heidelberg Germany; ^14^ Institute of Pathology University of Heidelberg Heidelberg Germany; ^15^ Department of Surgery, Division of Surgical Oncology and Gastrointestinal Surgery Radboud University Medical Center Nijmegen The Netherlands; ^16^ Department of General, Visceral and Transplantation Surgery University of Heidelberg Heidelberg Germany; ^17^ Department of Surgery Canisius‐Wilhelmina Hospital Nijmegen The Netherlands; ^18^ Department of Internal Medicine and Gastroenterology Zuyderland Medical Center Sittard The Netherlands; ^19^ Hospital Oberpullendorf Burgenland Austria; ^20^ BEVITAL Bergen Norway; ^21^ Department of Surgery VieCuri Medical Center Venlo The Netherlands; ^22^ Genome Biology European Molecular Biology Laboratory (EMBL) Heidelberg Germany

**Keywords:** colorectal cancer, disease stage, metabolomics, plasma metabolites, epidemiology

## Abstract

Colorectal cancer is the second most common cause of cancer‐related death globally, with marked differences in prognosis by disease stage at diagnosis. We studied circulating metabolites in relation to disease stage to improve the understanding of metabolic pathways related to colorectal cancer progression. We investigated plasma concentrations of 130 metabolites among 744 Stages I–IV colorectal cancer patients from ongoing cohort studies. Plasma samples, collected at diagnosis, were analyzed with liquid chromatography‐mass spectrometry using the Biocrates AbsoluteIDQ™ p180 kit. We assessed associations between metabolite concentrations and stage using multinomial and multivariable logistic regression models. Analyses were adjusted for potential confounders as well as multiple testing using false discovery rate (FDR) correction. Patients presented with 23, 28, 39 and 10% of Stages I–IV disease, respectively. Concentrations of sphingomyelin C26:0 were lower in Stage III patients compared to Stage I patients (*p*_FDR_ < 0.05). Concentrations of sphingomyelin C18:0 and phosphatidylcholine (diacyl) C32:0 were statistically significantly higher, while citrulline, histidine, phosphatidylcholine (diacyl) C34:4, phosphatidylcholine (acyl‐alkyl) C40:1 and lysophosphatidylcholines (acyl) C16:0 and C17:0 concentrations were lower in Stage IV compared to Stage I patients (*p*_FDR_ < 0.05). Our results suggest that metabolic pathways involving among others citrulline and histidine, implicated previously in colorectal cancer development, may also be linked to colorectal cancer progression.

## Abstract 2

**What's new?**

Metabolomics is a sophisticated method for investigating whether the metabolite profile of a patient's blood, etc., may reflect the pathophysiological state of cancers and other diseases. In the present study, the authors analyzed circulating metabolites, seeking biomarkers related to colorectal cancer progression. Their results at various stages of colorectal cancer suggest that metabolic pathways involving citrulline, histidine, and other molecules that have been previously implicated in colorectal cancer development may also be linked to progression.

**Abbreviations**
BMI: body mass index
CI: confidence interval
CORSA: Colorectal Cancer Study of Austria
FDR: false discovery rate
FOBT: fecal occult blood testing
IARC: International Agency for Research on Cancer
LOD: limit of detection
LysoPC a: lysophosphatidylcholine (acyl)
MS: mass spectrometry
N: lymph node
PC aa: phosphatidylcholine (diacyl)
PC ae: phosphatidylcholine (acyl‐alkyl)
SM: sphingomyelin
T: tumor

## Background

For colorectal cancer, disease stage at diagnosis is one of the key determinants of prognosis and survival.^1^, ^2^ To date, the mechanisms underlying cancer progression remain incompletely understood. In the current study, we aimed to study the association between circulating plasma metabolites at diagnosis and colorectal cancer stage.

Metabolomics is a sophisticated approach to measure concentrations of a large number of metabolites in biospecimens such as blood, and the metabolite profile may reflect the (patho)physiological state of individuals.^3^ Previous studies using metabolomics have been able to differentiate between individuals with and without colorectal cancer.^4^, ^5^, ^6^, ^7^, ^8^

However, few studies have investigated associations of circulating metabolites with colorectal cancer stage, and most existing studies^6^, ^9^, ^10^ generally had relatively small sample sizes. Farshidfar and colleagues used an untargeted approach to identify serum metabolites related to tumor (T) and lymph node (N) staging, among 178 patients with Stages I–III colorectal cancer.^6^ Thirteen out of the 40 metabolites differentiating T‐stages were known biological compounds, including pyruvate and the amino acids glutamine and lysine. Four out of the 17 metabolites differentiating N‐stages could be identified of which two were overlapping with metabolites distinguishing T‐stages (tocopherol and nonanoic acid). Glutamine and histidine concentrations were reported to be associated with T‐stage, both metabolites tended to be lowest among patients with advanced colorectal cancer.^11^ Another study showed a different serum metabolite profile, including differences in concentrations of several amino acids, between nonmetastatic colorectal cancer (Stages I–III, *n* = 42) *vs*. metastatic colorectal cancer with liver metastases (Stage IV, *n* = 45).^9^ In addition, another small study observed that pretreatment serum concentrations of deoxyinosine, pyridoxine, glycine, deoxycholic acid, taurocholic acid and cholesteryl esters were statistically significantly different between Stages 0–I (*n* = 8) and II–IV (*n* = 8) colorectal cancer patients.^10^

While these findings highlight the potential of metabolite profiling to identify circulating metabolites associated with colorectal cancer stage, these studies were limited by an inability to adjust for clinicodemographic and/or lifestyle factors such as age, sex, body mass index (BMI), tumor site and neoadjuvant therapy and differences in stage classification. Given that these factors influence metabolite concentrations^12^, ^13^, ^14^ and are involved in disease progression, this could potentially account for some differences seen in studies to date. Furthermore, prior analyses investigating metabolite profiles were often not corrected for multiple testing, increasing the chance of potential false‐positive findings.

Therefore, we investigated associations of plasma concentrations of metabolites and colorectal cancer stage in a large international consortium of four cohorts among colorectal cancer patients. We compared Stages II–IV individually with Stage I colorectal cancer, as well as differences between combined stages with and without lymph node and/or distant metastases (Stages III–IV *vs*. I–II).

## Methods

### Study design and populations

Data from four cohort studies within the MetaboCCC Consortium, a large consortium of European colorectal cancer cohorts established to investigate metabolic profiles across the continuum of colorectal cancer carcinogenesis, were included. The participating cohorts included: (*i*) the COLON study^15^ from the Netherlands (https://ClinicalTrials.gov Identifier: NCT03191110), (*ii*) the EnCoRe study^16^ from the Netherlands (Netherlands Trial Register: 7099), (*iii*) the Heidelberg, Germany, site of the international ColoCare Study^17^ (https://ClinicalTrials.gov Identifier: NCT02328677) and (*iv*) the Colorectal Cancer Study of Austria (CORSA). All cohorts were approved by local medical ethics committees and all participants provided written informed consent. In total, *n* = 744 newly diagnosed colorectal cancer patients were included in the current study (*n* = 197, 206, 285 and 56 from the COLON, EnCoRe, ColoCare and CORSA cohort, respectively).

**Figure 1:**
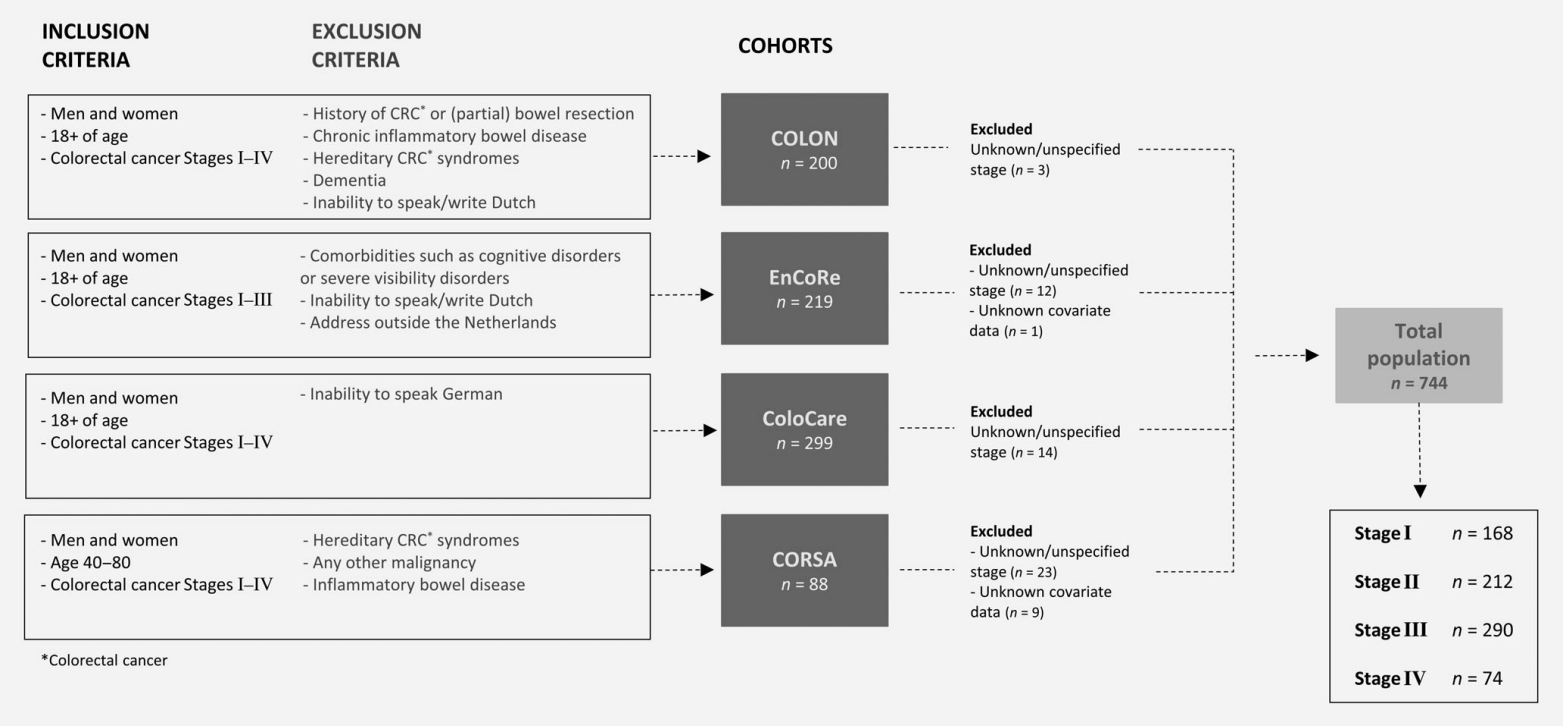
Flowchart of the total study population.

The inclusion and exclusion criteria of the individual cohorts are shown in Figure 1. Briefly, the COLON study is an ongoing prospective cohort study among colorectal cancer patients since 2010.^15^ Participants were recruited at time of diagnosis from 1 academic and 10 peripheral hospitals in the Netherlands.

The EnCoRe study, initiated in 2012, is an ongoing prospective cohort study.^16^ Colorectal cancer patients were recruited at diagnosis from one academic and two peripheral hospitals in the southeast of the Netherlands.

The ColoCare Study is an ongoing, international, multicenter prospective cohort study, which started in 2007.^17^ Colorectal cancer patients were recruited at the University Hospital of Heidelberg and the National Center for Tumor Diseases in Heidelberg, Germany.

CORSA is an ongoing study recruiting colorectal cancer patients in cooperation with the province‐wide screening project “Burgenland Prevention Trial of Colorectal Disease with Immunological Testing” (B‐PREDICT) since 2003, using fecal occult blood testing (FOBT). FOBT‐positive tested individuals subsequently received a complete colonoscopy. Additional colorectal cancer patients were recruited at diagnosis at four hospitals in Vienna.

All participants had histologically confirmed colorectal cancer and their plasma samples were assayed with a targeted metabolomics kit.

### Data collection

Across all cohorts, EDTA plasma samples were collected upon patient recruitment, that is, mostly shortly after colorectal cancer diagnosis and mostly before neoadjuvant therapy or surgery. Plasma samples were collected and processed within 4 hr and stored at the corresponding study sites at −80°C until further analysis.

Clinical data, including TNM‐stage, tumor site, and treatment regimen (i.e. surgery date, whether or not patients received neoadjuvant and adjuvant radio‐ and/or chemotherapy) were obtained from medical records for all cohorts. Both the pathological and clinical TNM characteristics, that is, pTNM and cTNM, were collected from medical records. pTNM was used for staging for patients with colon cancer undergoing primary tumor resection and for rectal cancer patients without neoadjuvant therapy, as neoadjuvant treatment may influence pTNM staging from tumor shrinkage to enable surgery.^18^ Therefore, cTNM was used to determine disease stage for all patients with neoadjuvant therapy as well as for colon cancer patients who did not undergo surgery, as pTNM staging is not available without tumor resection. Participants were staged to overall stage (I, II, III or IV) according to the TNM classification of Malignant Tumours of the Union for International Cancer Control (8^th^ version, 2016).^19^

Demographic and lifestyle characteristics, including age at diagnosis, sex, weight, height (to calculate BMI) and smoking status, were collected through study‐specific questionnaires, except for the EnCoRe study where weight and height were measured during home visits. Data for all clinical, demographic and lifestyle characteristics were harmonized across cohorts within the MetaboCCC Consortium.

### Biomarker analysis

All samples were shipped on dry ice and analyzed at the International Agency for Research on Cancer (IARC) in Lyon, France, using a targeted metabolomics kit (AbsoluteIDQ™ p180 kit: BIOCRATES Life Sciences AG, Innsbruck, Austria). COLON, EnCoRe and CORSA blood samples were thawed once while ColoCare samples were not thawed before shipment to IARC. All samples of each cohort were analyzed subsequently over a total of 19 batches between May and October 2016.

The analytical method characterizes up to *n* = 186 metabolites from five compound classes: *n* = 21 amino acids, *n* = 19 biogenic amines, *n* = 90 glycerophospholipids, *n* = 15 sphingolipids, *n* = 40 acylcarnitines and the sum of hexose sugars. The analytical procedure has previously been described in detail.^20^, ^21^ Briefly, liquid chromatography coupled to a tandem mass spectrometer was used, following the manufacturer's recommendations for sample preparation and analysis. Amino acids and biogenic amines were quantified by ultrahigh performance liquid chromatography coupled to tandem mass spectrometry (MS/MS), whereas lipids, sugar and acylcarnitines were semi‐quantified by flow injection analysis MS/MS.

Using quality control samples, metabolites with inter‐ or intra‐batch coefficients of variation >20% were excluded from further analysis. Subsequently, metabolites with >20% of missing values across all cohorts, including “true” missing values as well as values below the limit of detection (LOD), or outside quantitative range of the method were excluded. This approach resulted in a total of *n* = 130 metabolites for further investigation in the present study.

For these *n* = 130 metabolites, imputation for values outside measurable ranges was used in case of missing values (<20%) across all cohorts according to the following procedure. Values below the LOD were imputed by half of the batch‐specific LOD, while values below or above the quantitative range were imputed by the lower and upper limits of quantification, respectively, following a commonly used approach for these types of data.^21^, ^22^ An overview of all measured metabolites and the number of missing values can be found in Supplementary Table S1.

### Statistical analysis

Clinicodemographic and lifestyle characteristics were described for the total study population, by disease stage and by cohort using descriptive analyses. Smoking status was categorized as current smoker, former smoker or never smoker. Tumor site was characterized as colon (cecum, appendix and ascending colon, hepatic flexure, transverse colon, splenic flexure, descending colon and sigmoid colon) and rectal (rectosigmoid junction and rectum) cancer.

Plasma metabolite concentrations were log transformed using the natural logarithm to normalize distributions. Thereafter, metabolite concentrations were Z‐standardized to provide a better comparison of regression coefficients across metabolites.

Multinomial logistic regression models were computed to assess the associations between concentrations of the Z‐standardized metabolites separately as independent variables and for each colorectal cancer stage as dependent variable, using Stage I as reference. In addition, multivariable logistic regression models were used to evaluate associations between metabolites and combined disease stages with and without lymph node and/or distant metastases (Stages III–IV *vs*. I–II).

Analyses were adjusted for age at diagnosis (continuous), sex and BMI (continuous) as potential confounders based on published findings.^13^, ^14^ In addition, the Principal Component Partial R‐square analysis^23^ was conducted to estimate the total variability in plasma metabolite concentrations attributed to potential explanatory variables including tumor site, colorectal cancer stage, smoking status, age, analytical batch, BMI, sex and cohort; results can be found in Supplementary Figure S1. This was done as a supporting analysis to identify potential other confounders for our analysis next to the *a priori* defined confounders, age, sex and BMI and relevant technical factors cohort and analytical batch. As can be seen in Supplementary Figure S1, the variables age, sex, BMI, cohort and analytical batch explained ≥1% of the variability in metabolite levels, whereas tumor site and smoking status only explained <1% of the variability in metabolite concentrations. We further tested whether adjustment for smoking and tumor site changed effect estimates of our main analysis. Since changes were <10%, we decided not to include smoking status and tumor site as covariates in our models.

Residuals of each metabolite concentration were calculated using linear mixed models. Metabolite concentrations were used as the independent variable, whereas random intercepts for analytical batch nested within cohorts were considered the dependent variable, as has been applied previously.^22^, ^24^ This procedure was chosen since analytical batches were cohort specific; using a nested variable allows to adjust for individual cohort as well as analytical batch simultaneously. In addition, this method enabled a robust adjustment for batch and cohort, by calculating residuals of plasma metabolite concentrations that are independent of analytical batch and cohort influences. Subsequently, the residuals of plasma metabolite concentrations (continuous) were included as the independent variable, and separate (II, III or IV *vs*. I) or combined colorectal cancer stages (III–IV *vs*. I–II) was defined as the dependent variable in multinomial logistic regression models or multivariable logistic regression models, respectively.

Heterogeneity among cohorts was explored for the identified metabolites that were statistically significantly associated with individual disease stages. A random‐effects meta‐analysis approach^25^ was used and we evaluated the *I* ^2^ index as measure for heterogeneity.^26^, ^27^ Cohort‐specific risk estimates were calculated using multinomial logistic regression models. Individual colorectal cancer stages (with Stage I as reference) were added as independent variable and log transformed using the natural logarithm, Z‐standardized metabolite concentrations as dependent variable. Regression models were adjusted for sex, age, BMI (continuous) and analytical batch.

Additionally, to explore the influence of individual study cohorts, “leave‐one‐out” multinomial logistic regression analyses were conducted comparing individual colorectal cancer stages leaving out individual study cohorts one by one.

Stratified analyses were conducted to explore sex‐specific associations and differences in associations by tumor site, that is, colon compared to rectal cancer. Multiplicative interaction by the respective variables was tested using product terms in the logistic regression models. In addition, sensitivity analyses comparing individual stages were performed: (*i*) excluding patients from whom blood was not collected and processed on the same day (*n* = 82) and (*ii*) excluding patients from whom blood was collected during or after any type of treatment, that is, (neo‐) adjuvant chemotherapy and/or surgery (*n* = 140).

All statistical procedures were computed in R, version 3.3.6. False discovery rate (FDR)^28^ was used to correct for multiple testing and findings with an FDR‐corrected *p*‐value (*p*_FDR_) <0.05 were considered to be statistically significant.

## Results

### Study population characteristics

Characteristics for the total study population (*n* = 744) and by disease stage at diagnosis are shown in Table 1. Patients had a median age of 66 years (interquartile range: 59–73), the majority of patients were men (65%) and patients presented with Stage I (23%), II (28%), III (39%) or IV (10%) disease at diagnosis. Stage I patients had the highest median age and BMI compared to Stages II–IV colorectal cancer patients. Current smoking was reported by 11–17% of Stages I–IV patients. Among the study population, 58% had colon cancer, of which 28% proximal and 30% distal colon cancer and 42% had rectal cancer. The majority of Stage I colorectal cancer patients presented with distal colon cancer (42%), Stage II patients had the highest percentage of proximal colon cancer (41%) while Stages III and IV patients more often presented with rectal cancer, 57% and 42%, respectively. An overview of characteristics by cohort can be found in Supplementary Table S2, and metabolite concentrations by individual cohorts can be found in Supplementary Table S3.

**Table 1:** Baseline characteristics of the total study population and by disease stage

	Total population	Stage I^1^	Stage II^1^	Stage III^1^	Stage IV^1^
	*n* = 744	*n* = 168	*n* = 212	*n* = 290	*n* = 74
Male sex, *n* (%)	483 (64.9)	107 (63.7)	129 (60.8)	197 (67.9)	50 (67.6)
Age*, median ( interquartile range)	66.0 (59.0–73.0)	67.0 (61.0–73.0)	68.0 (62.0–74.0)	64.0 (57.0–72.0)	60.5 (51.0–70.0)
Body mass index, kg/m^2^					
**Median** (interquartile range)	26.5 (24.0–29.4)	27.1 (24.3–31.1)	26.2 (23.7–29.4)	26.5 (24.0–29.2)	25.8 (23.2–28.0)
**Underweight, <18.5** ***n*** (%)	10 (1.3)	0 (0)	3 (1.4)	4 (1.4)	3 (4.1)
**Normal weight, 18.5–24.9** *n* (%)	245 (32.9)	53 (31.5)	73 (34.4)	94 (32.4)	25 (33.8)
**Overweight, 25–29.9** *n* (%)	330 (44.4)	72 (42.9)	89 (42.0)	134 (46.2)	35 (47.3)
**Obese, ≥30** *n* (%)	159 (21.4)	43 (25.6)	47 (22.2)	58 (20.0)	11 (14.9)
Smoking status^2^, *n* (%)					
**Current**	111 (14.9)	18 (10.7)	35 (16.5)	48 (16.6)	10 (13.5)
**Former**	369 (49.6)	85 (50.6)	113 (53.3)	139 (47.9)	32 (43.2)
**Never**	244 (32.8)	59 (35.1)	60 (28.3)	94 (32.4)	31 (41.9)
Tumor site^3^^,^ 4 ^,^ *, *n* (%)					
**Colon – proximal**	205 (27.6)	42 (25.0)	86 (40.6)	55 (19.0)	22 (29.7)
**Colon – distal**	224 (30.1)	70 (41.7)	65 (30.7)	68 (23.4)	21 (28.4)
**Rectal**	313 (42.1)	55 (32.7)	61 (28.8)	166 (57.2)	31 (41.9)
Treatment, *n* (%)					
**Neoadjuvant therapy** *	208 (28.0)	19 (11.3)	30 (14.2)	130 (44.8)	29 (39.2)
**Surgery**	732 (98.4)	167 (99.4)	210 (99.1)	283 (97.6)	72 (97.3)

1 pTNM for patients who underwent surgery, cTNM for patients with rectal cancer who received neoadjuvant therapy and patients with colon cancer without surgery.

2 Missing data for 5, 13 and 1 patients of the EnCoRe, ColoCare and CORSA study, respectively.

3 Proximal consisting of: hepatic flexure, transverse colon, cecum, appendix, ascending colon; Distal consisting of: descending colon, sigmoid colon, splenic flexure; Rectal consisting of: rectosigmoid junction, rectum.

4 Missing data for 2 patients of the CORSA study.

* Statistically significant differences between stages (*p* ≤ 0.05) using Kruskal–Wallis tests or chi‐square tests.

### Associations of plasma metabolite concentrations with colorectal cancer stage

**Figure 2:**
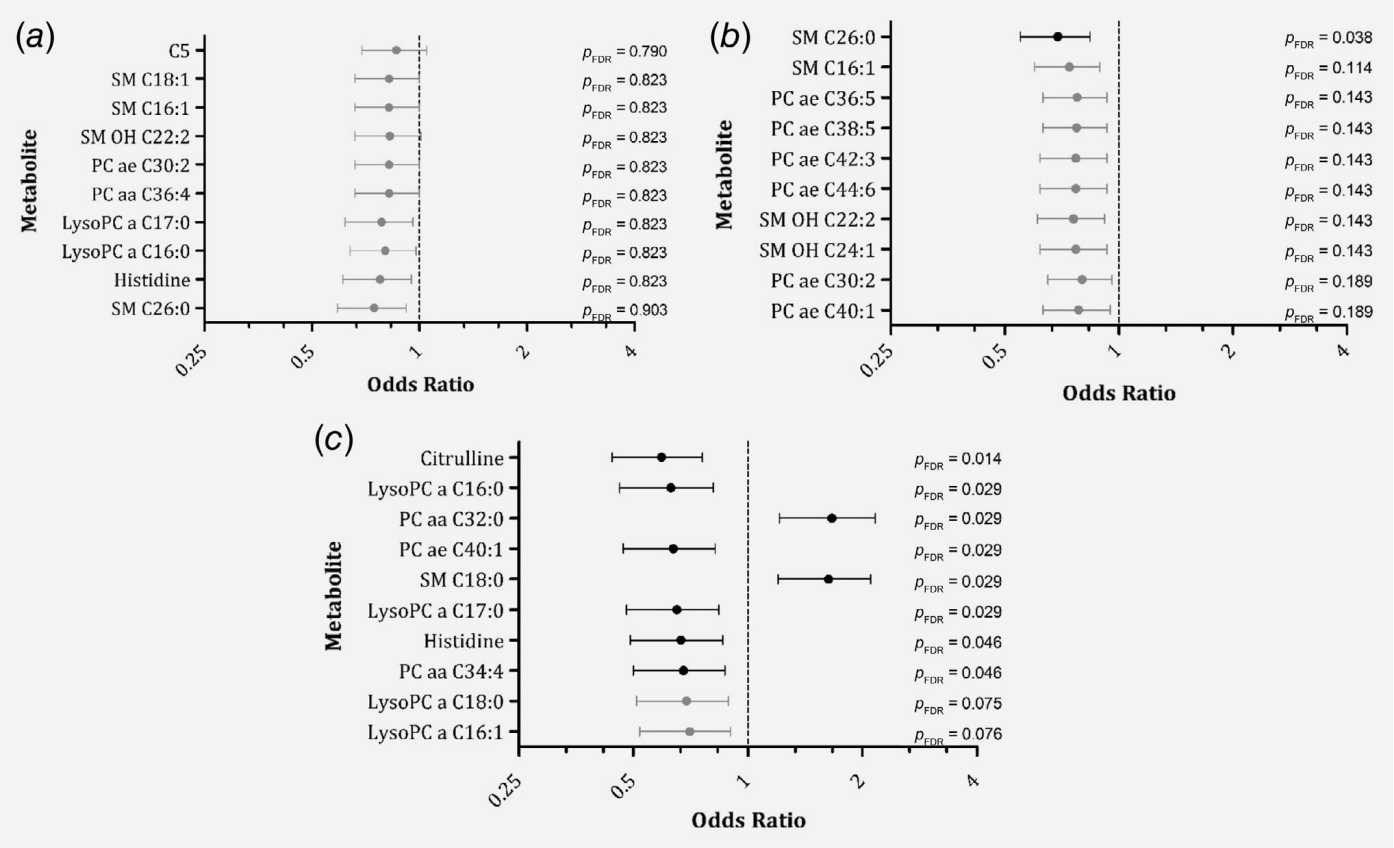
Top 10 plasma metabolites associated with colorectal cancer stages, ranked by *p*‐value. Black bars and symbols represent metabolites statistically significantly associated with stage after FDR correction (*p*_FDR_ ≤ 0.05). Gray bars and symbols represent metabolites not statistically significantly associated with stage after FDR correction (*p*_FDR_ > 0.05). (*a*). Top 10 plasma metabolites associated with Stage II (*n* = 212) colorectal cancer compared to Stage I (*n* = 168), ranked by *p*_FDR_. (*b*). Top 10 plasma metabolites associated with Stage III (*n* = 290) colorectal cancer compared to Stage I (*n* = 168), ranked by *p*_FDR_. (*c*) Top 10 plasma metabolites associated with Stage IV (*n* = 74) colorectal cancer compared to Stage I (*n* = 168), ranked by *p*_FDR_; Scale is logarithmic, *p*_FDR_: *p*‐value corrected for FDR.

Figure 2 illustrates the top 10 metabolites, ranked by *p*_FDR_, associated with Stage II, III or IV compared to Stage I, based on multinomial logistic regression analyses. When comparing Stage II with Stage I colorectal cancer, none of the metabolites were significantly associated with stage after correction for multiple testing, see Figure 2 *a*. Sphingomyelin (SM) C26:0 concentrations were lower among Stage III compared to Stage I patients (*p*_FDR_: 0.038), see Figure 2 *b*. Moreover, plasma concentrations of SM C18:0 and phosphatidylcholine (diacyl) (PC aa) C32:0 were statistically significantly higher, whereas citrulline, histidine, PC aa C34:4, phosphatidylcholine (acyl‐alkyl) (PC ae) C40:1 and lysophosphatidylcholines (acyl) (LysoPC a) C16:0 and C17:0 were statistically significantly lower among Stage IV colorectal cancer compared to Stage I patients after FDR correction, see Figure 2 *c*. Detailed results of all the included 130 metabolites by stage, including the mean and standard deviations of metabolite concentrations, can be found in Supplementary Table S4.

Heterogeneity among cohorts was evaluated for the identified metabolites that were found to be statistically significantly associated with colorectal cancer stage (as shown in Table 2). Overall, odds ratios (ORs) obtained from the random‐effects meta‐analysis showed comparable results with the ORs obtained from analysis within the total study population. Histidine showed high heterogeneity among cohorts (*I* ^2^ = 85.3%, *p* = 0.001).^29^ Both COLON and CORSA showed higher histidine concentrations in Stage IV colorectal cancer patients compared to Stage I patients (not statistically significant), while ColoCare showed an effect size in the opposite direction for histidine (statistically significant).

**Table 2:** Metabolites significantly associated with Stage III or IV compared to Stage I and the heterogeneity among cohorts^1^

Metabolite	Total study population	COLON	EnCoRe^2^	ColoCare	CORSA	Random‐effects meta‐analysis^3^
	OR (95% CI)	OR (95% CI)	OR (95% CI)	OR (95% CI)	OR (95% CI)	OR (95% CI)
Stage III compared to stage I						
Sphingomyelin C26:0	**0.68 (0.55–0.84)**	**0.63 (0.41–0.97)**	**0.60 (0.37–0.97)**	**0.59 (0.37–0.94)**	1.49 (0.63–3.53)	**0.67 (0.50–0.89)**
						(*I* ^2^ = 22.1%; *p* = 0.28)
Stage IV compared to stage I^4^						
Citrulline	**0.58 (0.44–0.76)**	0.53 (0.20–1.40)	–	**0.49 (0.31–0.78)**	0.82 (0.42–1.60)	**0.57 (0.40–0.81)**
						(*I* ^2^ = 0.0%; *p* = 0.45)
Lysophosphatidylcholine (acyl) C16:0	**0.61 (0.46–0.81)**	1.12 (0.30–4.15)	–	**0.64 (0.44–0.92)**	0.56 (0.19–1.66)	**0.65 (0.47–0.91)**
						(*I* ^2^ = 0.0%; *p* = 0.69)
Phosphatidylcholine (diacyl) C32:0	**1.62 (1.21–2.16)**	1.20 (0.46–3.18)	–	**1.68 (1.06–2.66)**	2.09 (0.96–4.52)	**1.68 (1.17–2.43)**
						(*I* ^2^ = 0.0%; *p* = 0.69)
Phosphatidylcholine (acyl‐alkyl) C40:1	**0.62 (0.47–0.82)**	0.58 (0.19–1.81)	–	**0.67 (0.46–0.99)**	0.39 (0.11–1.35)	**0.63 (0.45–0.90)**
						(*I* ^2^ = 0.0%; *p* = 0.71)
Sphingomyelin C18:0	**1.59 (1.20–2.10)**	0.66 (0.21–2.09)	–	**1.88 (1.18–3.01)**	**2.99 (1.15–7.75)**	1.70 (0.86–3.34)
						(*I* ^2^ = 50.6%; *p* = 0.13)
Lysophosphatidylcholine (acyl) C17:0	**0.63 (0.48–0.84)**	0.88 (0.25–3.08)	–	**0.70 (0.49–0.99)**	0.93 (0.37–2.33)	0.73 (0.53–1.01)
						(*I* ^2^ = 0.0%; *p* = 0.81)
Histidine	**0.65 (0.49–0.86)**	1.81 (0.42–7.70)	–	**0.30 (0.16–0.55)**	1.12 (0.73–1.72)	0.77 (0.27–2.22)
						(*I* ^2^ = 85.3%; *p* = 0.001)
Phosphatidylcholine (diacyl) C34:4	**0.66 (0.50–0.87)**	0.51 (0.16–1.59)	–	0.72 (0.49–1.07)	**0.68 (0.49–0.89)**	**0.69 (0.49–0.98)**
						(*I* ^2^ = 0.0%; *p* = 0.85)

Bold odds ratios (ORs) (95% CI) represent statistically significantly associated metabolites.

1 ORs per cohort were calculated using multinomial logistic regression models with colorectal cancer stage as independent variable (Stage I is reference) and log transformed Z‐standardized metabolite concentrations as dependent variable. Regression models were adjusted for sex, age, body mass index (continuous) and analytical batch. The use of residuals of metabolite concentrations in cohort‐specific analyses was not possible since there is no correction for cohort in this analysis. Therefore, batch was added as an additional confounder in the multinomial logistic regression model.

2 The EnCoRe study did not recruit Stage IV patients.

3 Heterogeneity among cohorts was evaluated using a random‐effects meta‐analysis approach and tested using the *I* ^2^ index with *p*‐value.

4 Ranked by *p*‐value of the main analysis.

“Leave‐one‐out” analyses, see Supplementary Table S5, showed comparable effects sizes and directions of the effect estimates compared to the analysis including the total study population. Excluding the EnCoRe study from analysis resulted in 15 and 3 additional statistically significant associated glycerophospholipids and sphingolipids, respectively, with colorectal cancer Stage III compared to Stage I, with ORs indicating somewhat stronger associations but with a similar direction of association as in the main analysis.

When evaluating associations between metabolite concentrations and Stages III‐IV compared to Stages I‐II, 15 out of 130 metabolites were statistically significantly associated with cancer stage prior to FDR correction, including 13 glycerophospholipids, SM C18:0 and citrulline. However, these associations were no longer statistically significant upon FDR correction. The 10 metabolites with the lowest *p*_FDR_ are presented in Table 3. Detailed results for metabolites comparing Stages III–IV *vs*. I–II can be found in Supplementary Table S6.

**Table 3:** Top 10 plasma metabolites associated with colorectal cancer stage (III–IV *vs*. I–II), ranked by *p*‐value^1^

	Metabolite	Abbreviation	Stages I–II^2^	Stages III–IV^2^	OR^3^	95% CI^4^	*p*‐value	*p*_FDR_ 5
			(*n* = 380)	(*n* = 364)				
			Mean ± SD	Mean ± SD				
1	Phosphatidylcholine (acyl‐alkyl) C42:3	PC ae C42:3	0.50 ± 0.14	0.47 ± 0.16	0.76	(0.65–0.89)	0.0008	0.099
2	Phosphatidylcholine (acyl‐alkyl) C40:1	PC ae C40:1	1.03 ± 0.30	0.96 ± 0.33	0.79	(0.68–0.93)	0.003	0.177
3	Phosphatidylcholine (acyl‐alkyl) C42:1	PC ae C42:1	0.30 ± 0.06	0.29 ± 0.07	0.79	(0.68–0.93)	0.004	0.177
4	Phosphatidylcholine (acyl‐alkyl) C44:6	PC ae C44:6	0.76 ± 0.22	0.72 ± 0.22	0.81	(0.69–0.94)	0.007	0.231
5	Phosphatidylcholine (diacyl) C42:2	PC aa C42:2	0.14 ± 0.04	0.13 ± 0.04	0.83	(0.71–0.97)	0.016	0.269
6	Phosphatidylcholine (acyl‐alkyl) C40:3	PC ae C40:3	0.83 ± 0.19	0.80 ± 0.21	0.82	(0.70–0.97)	0.017	0.269
7	Sphingomyelin C18:0	SM C18:0	13.55 ± 3.78	14.44 ± 4.74	1.21	(1.03–1.42)	0.018	0.269
8	Phosphatidylcholine (acyl‐alkyl) C38:5	PC ae C38:5	21.64 ± 4.98	20.63 ± 5.34	0.84	(0.72–0.97)	0.019	0.269
9	Phosphatidylcholine (acyl‐alkyl) C44:3	PC ae C44:3	0.06 ± 0.02	0.06 ± 0.02	0.83	(0.71–0.97)	0.020	0.269
10	Phosphatidylcholine (acyl‐alkyl) C34:3	PC ae C34:3	11.99 ± 4.12	11.27 ± 4.14	0.83	(0.72–0.97)	0.021	0.269

1 Analyzed using multiple logistic regression models analyzing associations of colorectal cancer stage (III–IV *vs*. I–II) as independent variable and the residuals obtained from linear mixed models with log transformed Z‐standardized metabolite concentrations as dependent variable with random intercepts for analytical batch nested within cohort. Regression models were adjusted for sex, age and body mass index (continuous).

2 Untransformed and unadjusted metabolite concentrations in μmol/l.

3 Odds ratio (e^β^), for Stages III–IV *vs*. I–II colorectal cancer per SD increase in transformed metabolite concentrations.

4 Confidence interval.

5 *p‐*value corrected for false discovery rate.

Results of the stratified analyses by sex and tumor site comparing individual diseases stages showed similar results compared to the analysis within the total study population (data not shown). No significant interactions by sex and tumor site were observed. Sensitivity analysis, (*i*) excluding patients from whom blood was not collected and processed on the same day (*n* = 82) and (*ii*) excluding patients from whom blood was collected during or after any type of treatment, that is, (neo‐) adjuvant chemotherapy and/or surgery (*n* = 140), did not influence the effect sizes compared to the main analysis (data not shown). Histidine was not statistically significantly associated with Stage IV compared to Stage I in both sensitivity analyses. Furthermore, SM C26:0 was borderline significant in both sensitivity analyses when comparing Stage III with Stage I (both *p*_FDR_: 0.065). Lastly, both sensitivity analyses showed some additional statistically significantly associated glycerophospholipids with Stage IV compared to Stage I. Similar to the “leave‐one‐out” analyses, somewhat stronger associations were observed in the sensitivity analyses but with a similar direction of association as in the main analysis.

## Discussion

Associations between plasma metabolites and colorectal cancer stage were investigated in a large international consortium of four ongoing cohorts among colorectal cancer patients. We observed lower concentrations of SM C26:0 in patients with Stage III compared to Stage I colorectal cancer. Plasma concentrations of SM C18:0 and PC aa C32:0 were higher in Stage IV patients compared to Stage I patients. Citrulline, histidine, PC aa C34:4, PC ae C40:1 and LysoPC a C16:0 and C17:0 showed lower plasma concentrations in Stage IV compared to Stage I patients. No statistically significant associations were observed between metabolites and combined stages with and without lymph node and/or distant metastases (Stages III–IV *vs*. I–II).

We made the novel observation that plasma concentrations of citrulline were lower in Stage IV compared to Stage I colorectal cancer patients. Previously, damage in the small intestine was reported to be reflected by decreased concentrations of circulating citrulline.^30^ Furthermore, citrulline plays a role in the urea cycle,^31^ previously linked to colorectal cancer development.^24^ In addition, histidine concentrations were also lower among Stage IV compared to Stage I colorectal cancer patients in the current study. Histidine is linked to aspartate metabolism and is one of the amino acids entering the tricarboxylic acid cycle.^32^ The tricarboxylic cycle has been reported in colorectal cancer development, as differences have been found comparing colorectal tumor tissue with normal mucosa.^5^, ^33^ Differences in serum histidine were observed among 336 newly diagnosed colorectal cancer patients; histidine tented to be lowest among advanced colorectal cancer patients. Investigators reported systemic inflammation to be an important determinant of histidine concentrations.^11^ Decreased citrulline and histidine concentrations among Stage IV colorectal cancer may also result from increased hepatic uptake^34^ due to possible liver metastases.

Next to histidine and citrulline, plasma PC aa C34:4, PC ae C40:1 and LysoPC a C16:0 and C17:0 also showed lower concentrations in Stage IV patients compared to Stage I colorectal cancer patients, while SM C18:0 and PC aa C32:0 showed higher concentrations. In addition, lower plasma concentrations of SM C26:0 were found in Stage III compared to Stage I patients. These seven metabolites have not been associated with colorectal cancer in previous studies. Replication of our results is warranted for verification and to establish a broader understanding of metabolites and potential pathways involved in colorectal cancer progression.

Comparison of our results with previous studies investigating the relationship between metabolites and colorectal cancer stage is difficult as not many studies have been done to date, and the existing studies compared different combinations of stages (e.g. Stages 0–I *vs*. II–IV) and used different sample matrices (serum or urine).^35^ In addition, most studies did not adjust for potential confounding factors^9^, ^10^, ^11^ or multiple testing^7^ and had a small sample size.^6^, ^9^, ^10^ Furthermore, previous studies mainly used untargeted metabolomics or targeted methods covering different metabolites than in the current study.

An important strength of the current study is that we were able to investigate associations between circulating metabolites and distinct colorectal cancer stages in a large cohort of colorectal cancer patients included in the MetaboCCC consortium, with availability of harmonized data on potential confounders including clinicodemographic and lifestyle characteristics (e.g., BMI). In addition, we were able to conduct a robust correction for analytical batch and individual cohorts using the residuals of metabolite concentrations adjusted for batch nested in cohort.

It should be noted that our findings, especially for histidine, warrant careful interpretation and replication as the number of participants for some stages of disease were relatively limited (especially for Stage IV with 74 patients) and not all cohorts recruited Stage IV patients. When leaving out the EnCoRe study, more statistically significant associations were observed with Stage III compared to Stage I colorectal cancer. The results indicated somewhat stronger associations but with a similar direction of association as in the main analysis. In addition, means and standard deviations of these metabolites did not differ between the cohorts. Baseline characteristics, study design or sample collection characteristics could not explain the observed differences, since these were similar between cohorts. The EnCoRe study contributes one third of Stage III patients, and excluding this cohort, therefore, likely has a substantial influence on results. We have no further biological explanation. Still, overall heterogeneity among cohorts for the reported metabolites appeared to be minimal, except for histidine, which showed effect sizes in the opposite direction for individual cohorts. However, the findings may provide important clues for further studies focusing on mechanisms of colorectal cancer progression. Alcohol intake and fasting status have been suggested to potentially influence the concentration of certain metabolites, such as acylcarnitines and phosphatidylcholines.^21^, ^22^, ^36^ We were not able to take these factors into account, because of incomplete or unavailable data in our cohorts. The issue of reverse causality should also be considered in our study because disease‐related lifestyle behaviors or advanced tumor characteristics, such as lymph node involvement or disturbed liver metabolism resulting from metastases, may also be responsible for altered plasma metabolite profiles.

In conclusion, we reported associations in plasma metabolite concentrations when comparing Stage III and Stage IV patients individually with patients diagnosed with Stage I disease, including amino acids, sphingolipids and glycerophospholipids. Our findings suggest that key metabolic pathways involving among other citrulline and histidine, implicated in colorectal carcinogenesis in previous studies, may be linked to disease progression as well. Further research in large sample sizes is warranted to replicate our findings, including other metabolite profiling methods, utilizing either targeted or untargeted metabolomics in different sample matrices to provide further insight into the metabolic pathways associated with colorectal cancer progression.

## Ethics approval and consent to participate

The study was performed in accordance with the Declaration of Helsinki. The COLON study has been approved by the Committee on Research involving Human Subjects (region Arnhem‐Nijmegen), the Netherlands. The EnCoRe study has been approved by the Medical Ethics Committee of the University Hospital Maastricht and Maastricht University, the Netherlands. The ColoCare Study has been approved by the ethics committee of the Medical Faculty at the University of Heidelberg. The CORSA Study has been approved by the institutional review board “Ethikkommission Burgenland” and by the ethical review committee of the Medical University of Vienna. All patients included in the analysis provided a written informed consent.

## Supporting information


**Supplementary Table S1** Metabolites measured in the BIOCRATES Absolute IDQ^TM^ p180 kit in the total study population and by individual cohort, and whether the metabolite was included for the current analysis

**Supplementary Table S2** Baseline clinicodemographic and lifestyle characteristics of the total study population and by individual cohort

**Supplementary Table S3** Plasma metabolite concentrations by individual cohort

**Supplementary Table S4** Results of plasma metabolites in the total study population using multinomial analysis comparing stage II, III and IV *vs*. stage I colorectal cancer

**Supplementary Table S5** Results of the “Leave‐one‐out” multinomial analyses comparing stage II, III and IV *vs*. stage I colorectal cancer

**Supplementary Table S6** Results of plasma metabolites associated with colorectal cancer stage (III‐IV *vs*. I‐II) in the total study population

**Supplementary Figure S1** Weighted and overall R_partial_^2^ for covariates showing the percentage of variability explained by each covariate separately and combined in metabolite concentrations, from the PC‐PR2 analysis. Included covariates were stage (I/II/III/IV), smoking status (current/former/never), tumor site (distal/proximal/rectal), analytical batch (1‐19), sex, cohort (COLON/EnCoRe/ColoCare/ CORSA), age and body mass index (continuous).

## Data Availability

The data sets used and/or analyzed during the current study are available from the corresponding author on reasonable request.
