# Supplementary material for: Plasma metabolites associated with colorectal cancer stage: Findings from an international consortium
Source: Int J Cancer. 2019 Oct 10;146(12):3256–66. doi: 10.1002/ijc.32666 (PMC7216900; doi:10.1002/ijc.32666)
Supplement: Supplementary file 1 — Supplementary Table S1 Metabolites measured in the BIOCRATES Absolute IDQTM p180 kit in the total study population and by individual cohort, and whether the metabolite was included for the current analysis [file IJC-146-3256-s001.docx]

**Supplementary Table S1**. Metabolites measured in the BIOCRATES Absolute IDQTM p180 kit in the total study population and by individual cohort, and whether the metabolite was included for the current analysis

|  |  |  | | | | | |  | **COHORTS** | | | | | | | |
| --- | --- | --- | --- | --- | --- | --- | --- | --- | --- | --- | --- | --- | --- | --- | --- | --- |
|  |  | **Total population (n= 744)** | | | | | |  | COLON (n=197) | | EnCoRe (n=206) | | ColoCare (n=285) | | CORSA (n=56) | |
| *Biochemical name* | *Abbreviation* | *Detec ted in ≥1 cohort* | *% missing* | *% <LOD or <LOQ** | *% >ULOD*** | *Total % missing /outside limits* | ***Incl. for analysis*** |  | *Detected* | *Total % missing /outside limits* | *Detected* | *Total % missing /outside limits* | *Detected* | *Total % missing /outside limits* | *Detected* | *Total % missing /outside limits* |
| **ACYLCARNITINES** |  |  |  |  |  |  |  |  |  |  |  |  |  |  |  |  |
| Carnitine | C0 | yes | 0.0 | 0.0 | 0.0 | 0.0 | **yes** |  | yes | 0.0 | yes | 0.0 | yes | 0.0 | yes | 0.0 |
| Acetylcarnitine | C2 | yes | 0.0 | 0.0 | 0.0 | 0.0 | **yes** |  | yes | 0.0 | yes | 0.0 | yes | 0.0 | yes | 0.0 |
| Propionylcarnitine | C3 | yes | 0.0 | 0.0 | 0.0 | 0.0 | **yes** |  | yes | 0.0 | yes | 0.0 | yes | 0.0 | yes | 0.0 |
| Propenoylcarnitine | C3:1 | no | 100.0 | 0.0 | 0.0 | 100.0 | **no** |  | no | 100.0 | no | 100.0 | no | 100.0 | no | 100.0 |
| Hydroxypropionaylcarnitine | C3-OH | no | 100.0 | 0.0 | 0.0 | 100.0 | **no** |  | no | 100.0 | no | 100.0 | no | 100.0 | no | 100.0 |
| Butyrylcarnitine | C4 | yes | 0.0 | 0.1 | 0.0 | 0.1 | **yes** |  | yes | 0.0 | yes | 0.0 | yes | 0.4 | yes | 0.0 |
| Butenylcarnitine | C4:1 | no | 100.0 | 0.0 | 0.0 | 100.0 | **no** |  | no | 100.0 | no | 100.0 | no | 100.0 | no | 100.0 |
| Hydroxybutyrylcarnitine | C4-OH (C3-DC) | yes | 0.0 | 50.5 | 0.0 | 50.5 | **no** |  | yes | 45.6 | yes | 70.9 | yes | 47.7 | yes | 17.9 |
| Valerylcarnitine | C5 | yes | 0.0 | 3.2 | 0.0 | 3.2 | **yes** |  | yes | 3.6 | yes | 1.9 | yes | 3.5 | yes | 5.4 |
| Tiglylcarnitine | C5:1 | no | 100.0 | 0.0 | 0.0 | 100.0 | **no** |  | no | 100.0 | no | 100.0 | no | 100.0 | no | 100.0 |
| Glutarylcarnitine | C5-DC (C6-OH) | yes | 92.5 | 5.5 | 0.0 | 98.0 | **no** |  | no | 100.0 | no | 100.0 | no | 100.0 | yes | 73.2 |
| Glutaconylcarnitine | C5-1-Dc | no | 100.0 | 0.0 | 0.0 | 100.0 | **no** |  | no | 100.0 | no | 100.0 | no | 100.0 | no | 100.0 |
| Methylglutarylcarnitine | C5-M-Dc | no | 100.0 | 0.0 | 0.0 | 100.0 | **no** |  | no | 100.0 | no | 100.0 | no | 100.0 | no | 100.0 |
| Hydroxyvalerylcarnitine | C5-OH (C3-DC-M) | no | 100.0 | 0.0 | 0.0 | 100.0 | **no** |  | no | 100.0 | no | 100.0 | no | 100.0 | no | 100.0 |
| Hexanoylcarnitine | C6 (C4:1-DC) | no | 100.0 | 0.0 | 0.0 | 100.0 | **no** |  | no | 100.0 | no | 100.0 | no | 100.0 | no | 100.0 |
| Hexenoylcarnitine | C6:1 | no | 100.0 | 0.0 | 0.0 | 100.0 | **no** |  | no | 100.0 | no | 100.0 | no | 100.0 | no | 100.0 |
| Pimelylcarnitine | C7-DC | yes | 64.8 | 31.3 | 0.0 | 96.1 | **no** |  | no | 100.0 | yes | 92.7 | no | 100.0 | yes | 75.0 |
| Octanoylcarnitine | C8 | yes | 0.0 | 79.6 | 0.0 | 79.6 | **no** |  | yes | 85.8 | yes | 85.4 | yes | 77.5 | yes | 46.4 |
| Nonaylcarnitine | C9 | yes | 0.0 | 86.2 | 0.0 | 86.2 | **no** |  | yes | 74.1 | yes | 85.9 | yes | 94.0 | yes | 89.3 |
| Decanoylcarnitine | C10 | yes | 0.0 | 50.9 | 0.0 | 50.9 | **no** |  | yes | 53.3 | yes | 52.9 | yes | 56.5 | yes | 7.1 |
| Decenoylcarnitine | C10:1 | yes | 0.0 | 24.5 | 0.0 | 24.5 | **no** |  | yes | 15.7 | yes | 18.9 | yes | 37.9 | yes | 7.1 |
| Decadienylcarnitine | C10:2 | no | 100.0 | 0.0 | 0.0 | 100.0 | **no** |  | no | 100.0 | no | 100.0 | no | 100.0 | no | 100.0 |
| Dodecanoylcarnitine | C12 | yes | 0.0 | 34.5 | 0.0 | 34.5 | **no** |  | yes | 31.5 | yes | 65.0 | yes | 19.6 | yes | 8.9 |
| Dodecenoylcarnitine | C12:1 | yes | 0.0 | 15.5 | 0.0 | 15.5 | **yes** |  | yes | 36.0 | yes | 1.9 | yes | 13.0 | yes | 5.4 |
| Dodecanedioylcarnitine | C12-DC | no | 100.0 | 0.0 | 0.0 | 100.0 | **no** |  | no | 100.0 | no | 100.0 | no | 100.0 | no | 100.0 |
| Tetradecanoylcarnitine | C14 | yes | 0.0 | 31.3 | 0.0 | 31.3 | **no** |  | yes | 22.3 | yes | 69.4 | yes | 14.4 | yes | 8.9 |
| Tetradecenoylcarnitine | C14:1 | yes | 0.0 | 0.1 | 0.0 | 0.1 | **yes** |  | yes | 0.0 | yes | 0.0 | yes | 0.4 | yes | 0.0 |
| Hydroxytetradecenoyl-carnitine | C14:1-OH | no | 100.0 | 0.0 | 0.0 | 100.0 | **no** |  | no | 100.0 | no | 100.0 | no | 100.0 | no | 100.0 |
| Tetradecadienylcarnitine | C14:2 | yes | 0.0 | 23.1 | 0.0 | 23.1 | **no** |  | yes | 11.2 | yes | 39.8 | yes | 23.2 | yes | 3.6 |
| Hydroxytetradecadienyl-carnitine | C14:2-OH | no | 100.0 | 0.0 | 0.0 | 100.0 | **no** |  | no | 100.0 | no | 100.0 | no | 100.0 | no | 100.0 |
| Hexadecanoylcarnitine | C16 | yes | 0.0 | 0.0 | 0.0 | 0.0 | **yes** |  | yes | 0.0 | yes | 0.0 | yes | 0.0 | yes | 0.0 |
| Hexadecenoylcarnitine | C16:1 | yes | 0.0 | 66.3 | 0.0 | 66.3 | **no** |  | yes | 83.2 | yes | 85.0 | yes | 48.8 | yes | 26.8 |
| Hydroxyhexadecenoylcarnitine | C16:1-OH | no | 100.0 | 0.0 | 0.0 | 100.0 | **no** |  | no | 100.0 | no | 100.0 | no | 100.0 | no | 100.0 |
| Hexadecadienylcarnitine | C16:2 | no | 100.0 | 0.0 | 0.0 | 100.0 | **no** |  | no | 100.0 | no | 100.0 | no | 100.0 | no | 100.0 |
| Hydroxyhexadecadienyl-carnitine | C16:2-OH | no | 100.0 | 0.0 | 0.0 | 100.0 | **no** |  | no | 100.0 | no | 100.0 | no | 100.0 | no | 100.0 |
| Hydroxyhexadecanoylcarnitine | C16-OH | no | 100.0 | 0.0 | 0.0 | 100.0 | **no** |  | no | 100.0 | no | 100.0 | no | 100.0 | no | 100.0 |
| Octadecanoylcarnitine | C18 | yes | 0.0 | 1.1 | 0.0 | 1.1 | **yes** |  | yes | 0.5 | yes | 0.0 | yes | 2.5 | yes | 0.0 |
| Octadecenoylcarnitine | C18:1 | yes | 0.0 | 0.0 | 0.0 | 0.0 | **yes** |  | yes | 0.0 | yes | 0.0 | yes | 0.0 | yes | 0.0 |
| Hydroxyoctadecenoylcarnitine | C18:1-OH | no | 100.0 | 0.0 | 0.0 | 100.0 | **no** |  | no | 100.0 | no | 100.0 | no | 100.0 | no | 100.0 |
| Octadecadienylcarnitine | C18:2 | yes | 0.0 | 0.0 | 0.0 | 0.0 | **yes** |  | yes | 0.0 | yes | 0.0 | yes | 0.0 | yes | 0.0 |
| **AMINO ACIDS** |  |  |  |  |  |  |  |  |  |  |  |  |  |  |  |  |
| Alanine | Ala | yes | 0.5 | 0.0 | 0.0 | 0.5 | **yes** |  | yes | 0.0 | yes | 0.0 | yes | 0.0 | yes | 7.1 |
| Arginine | Arg | yes | 0.5 | 0.3 | 0.0 | 0.8 | **yes** |  | yes | 0.0 | yes | 0.0 | yes | 0.7 | yes | 7.1 |
| Asparagine | Asn | yes | 0.5 | 0.0 | 0.0 | 0.5 | **yes** |  | yes | 0.0 | yes | 0.0 | yes | 0.0 | yes | 7.1 |
| Aspartate | Asp | yes | 0.5 | 16.3 | 0.0 | 16.8 | **yes** |  | yes | 5.1 | yes | 4.9 | yes | 31.9 | yes | 25.0 |
| Citrulline | Cit | yes | 0.5 | 0.0 | 0.0 | 0.5 | **yes** |  | yes | 0.0 | yes | 0.0 | yes | 0.0 | yes | 7.1 |
| Glutamine | Gln | yes | 0.5 | 0.0 | 0.0 | 0.5 | **yes** |  | yes | 0.0 | yes | 0.0 | yes | 0.0 | yes | 7.1 |
| Glutamate | Glu | yes | 0.5 | 0.0 | 0.0 | 0.5 | **yes** |  | yes | 0.0 | yes | 0.0 | yes | 0.0 | yes | 7.1 |
| Glycine | Gly | yes | 0.5 | 0.0 | 0.0 | 0.5 | **yes** |  | yes | 0.0 | yes | 0.0 | yes | 0.0 | yes | 7.1 |
| Histidine | His | yes | 0.5 | 0.0 | 0.0 | 0.5 | **yes** |  | yes | 0.0 | yes | 0.0 | yes | 0.0 | yes | 7.1 |
| Isoleucine | Ile | yes | 0.5 | 0.0 | 0.0 | 0.5 | **yes** |  | yes | 0.0 | yes | 0.0 | yes | 0.0 | yes | 7.1 |
| Leucine | Leu | yes | 0.5 | 0.3 | 0.0 | 0.8 | **yes** |  | yes | 0.5 | yes | 0.0 | yes | 0.4 | yes | 7.1 |
| Lysine | Lys | yes | 0.5 | 0.0 | 0.0 | 0.5 | **yes** |  | yes | 0.0 | yes | 0.0 | yes | 0.0 | yes | 7.1 |
| Methionine | Met | yes | 0.5 | 0.2 | 0.0 | 0.7 | **yes** |  | yes | 0.0 | yes | 0.0 | yes | 0.4 | yes | 7.1 |
| Ornithine | Orn | yes | 0.5 | 0.0 | 0.0 | 0.5 | **yes** |  | yes | 0.0 | yes | 0.0 | yes | 0.0 | yes | 7.1 |
| Phenylalanine | Phe | yes | 0.5 | 0.0 | 0.0 | 0.5 | **yes** |  | yes | 0.0 | yes | 0.0 | yes | 0.0 | yes | 7.1 |
| Proline | Pro | yes | 0.5 | 0.0 | 0.0 | 0.5 | **yes** |  | yes | 0.0 | yes | 0.0 | yes | 0.0 | yes | 7.1 |
| Serine | Ser | yes | 0.5 | 0.0 | 0.0 | 0.5 | **yes** |  | yes | 0.0 | yes | 0.0 | yes | 0.0 | yes | 7.1 |
| Threonine | Thr | yes | 0.5 | 0.0 | 0.0 | 0.5 | **yes** |  | yes | 0.0 | yes | 0.0 | yes | 0.0 | yes | 7.1 |
| Tryptophan | Trp | yes | 0.5 | 0.0 | 0.0 | 0.5 | **yes** |  | yes | 0.0 | yes | 0.0 | yes | 0.0 | yes | 7.1 |
| Tyrosine | Tyr | yes | 0.5 | 0.0 | 0.0 | 0.5 | **yes** |  | yes | 0.0 | yes | 0.0 | yes | 0.0 | yes | 7.1 |
| Valine | Val | yes | 0.5 | 0.0 | 0.0 | 0.5 | **yes** |  | yes | 0.0 | yes | 0.0 | yes | 0.0 | yes | 7.1 |
| **BIOGENIC AMINES** |  |  |  |  |  |  |  |  |  |  |  |  |  |  |  |  |
| Acetylornithine | Ac-Orn | no | 100.0 | 0.0 | 0.0 | 100.0 | **no** |  | no | 100.0 | no | 100.0 | no | 100.0 | no | 100.0 |
| alpha-Aminoadipic acid | alpha-AAA | yes | 0.5 | 62.8 | 0.0 | 63.3 | **no** |  | yes | 54.8 | yes | 59.7 | yes | 73.0 | yes | 57.1 |
| Carnosine | Carnosine | no | 100.0 | 0.0 | 0.0 | 100.0 | **no** |  | no | 100.0 | no | 100.0 | no | 100.0 | no | 100.0 |
| Creatinine | Creatinine | yes | 0.5 | 0.0 | 0.0 | 0.5 | **yes** |  | yes | 0.0 | yes | 0.0 | yes | 0.0 | yes | 7.1 |
| Histamine | Histamine | no | 100.0 | 0.0 | 0.0 | 100.0 | **no** |  | no | 100.0 | no | 100.0 | no | 100.0 | no | 100.0 |
| Kynurenine | Kynurenine | yes | 0.5 | 0.6 | 0.0 | 1.1 | **yes** |  | yes | 0.0 | yes | 0.0 | yes | 1.4 | yes | 7.1 |
| Methioninesulfoxide | Met-SO | no | 100.0 | 0.0 | 0.0 | 100.0 | **no** |  | no | 100.0 | no | 100.0 | no | 100.0 | no | 100.0 |
| Nitrotyrosine | Nitro-Tyr | no | 100.0 | 0.0 | 0.0 | 100.0 | **no** |  | no | 100.0 | no | 100.0 | no | 100.0 | no | 100.0 |
| Phenylethylamine | PEA | no | 100.0 | 0.0 | 0.0 | 100.0 | **no** |  | no | 100.0 | no | 100.0 | no | 100.0 | no | 100.0 |
| Putrescine | Putrescine | yes | 0.5 | 36.6 | 0.0 | 37.1 | **no** |  | yes | 29.4 | yes | 24.3 | yes | 52.3 | yes | 33.9 |
| Sarcosine | Sarcosine | yes | 0.5 | 0.2 | 0.0 | 0.7 | **yes** |  | yes | 0.0 | yes | 0.0 | yes | 0.4 | yes | 7.1 |
| Serotonin | Serotonin | yes | 0.5 | 44.3 | 0.0 | 44.8 | **no** |  | yes | 8.1 | yes | 7.3 | yes | 96.1 | yes | 50.0 |
| Spermidine | Spermidine | yes | 0.5 | 68.7 | 0.0 | 69.2 | **no** |  | yes | 56.3 | yes | 73.3 | yes | 77.5 | yes | 57.1 |
| Spermine | Spermine | yes | 0.5 | 79.7 | 0.0 | 80.2 | **no** |  | yes | 76.6 | yes | 62.6 | yes | 94.0 | yes | 87.5 |
| Taurine | Taurine | yes | 17.5 | 0.0 | 3.5 | 21.0 | **no** |  | yes | 10.7 | yes | 1.0 | yes | 0.0 | yes | 12.5 |
| Total dimethylarginine | Total DMA | no | 100.0 | 0.0 | 0.0 | 100.0 | **no** |  | no | 100.0 | no | 100.0 | no | 100.0 | no | 100.0 |
| Trans 4-Hydroxyproline | t4-OH-Pro | yes | 0.5 | 0.0 | 0.0 | 0.5 | **yes** |  | yes | 0.0 | yes | 0.0 | yes | 0.0 | yes | 7.1 |
| Asymmetric dimethylarginine | ADMA | yes | 0.5 | 0.8 | 0.0 | 1.3 | **yes** |  | yes | 1.0 | yes | 0.0 | yes | 0.7 | yes | 10.7 |
| Symmetric dimethylarginine | SDMA | yes | 0.5 | 0.0 | 0.0 | 0.5 | **yes** |  | yes | 0.0 | yes | 0.0 | yes | 0.0 | yes | 7.1 |
| **GLYCEROPHOSPHOLIPIDS** |  |  |  |  |  |  |  |  |  |  |  |  |  |  |  |  |
| Lysophosphatidylcholine (acyl) C14:0 | lysoPC a C14:0 | no | 100.0 | 0.0 | 0.0 | 100.0 | **no** |  | no | 100.0 | no | 100.0 | no | 100.0 | no | 100.0 |
| Lysophosphatidylcholine (acyl) C16:0 | lysoPC a C16:0 | yes | 0.0 | 0.0 | 0.0 | 0.0 | **yes** |  | yes | 0.0 | yes | 0.0 | yes | 0.0 | yes | 0.0 |
| Lysophosphatidylcholine (acyl) C16:1 | lysoPC a C16:1 | yes | 0.0 | 0.0 | 0.0 | 0.0 | **yes** |  | yes | 0.0 | yes | 0.0 | yes | 0.0 | yes | 0.0 |
| Lysophosphatidylcholine (acyl) C17:0 | lysoPC a C17:0 | yes | 0.0 | 0.0 | 0.0 | 0.0 | **yes** |  | yes | 0.0 | yes | 0.0 | yes | 0.0 | yes | 0.0 |
| Lysophosphatidylcholine (acyl) C18:0 | lysoPC a C18:0 | yes | 0.0 | 0.0 | 0.0 | 0.0 | **yes** |  | yes | 0.0 | yes | 0.0 | yes | 0.0 | yes | 0.0 |
| Lysophosphatidylcholine (acyl) C18:1 | lysoPC a C18:1 | yes | 0.0 | 0.0 | 0.0 | 0.0 | **yes** |  | yes | 0.0 | yes | 0.0 | yes | 0.0 | yes | 0.0 |
| Lysophosphatidylcholine (acyl) C18:2 | lysoPC a C18:2 | yes | 0.0 | 0.0 | 0.0 | 0.0 | **yes** |  | yes | 0.0 | yes | 0.0 | yes | 0.0 | yes | 0.0 |
| Lysophosphatidylcholine (acyl) C20:3 | lysoPC a C20:3 | yes | 0.0 | 0.0 | 0.0 | 0.0 | **yes** |  | yes | 0.0 | yes | 0.0 | yes | 0.0 | yes | 0.0 |
| Lysophosphatidylcholine (acyl) C20:4 | lysoPC a C20:4 | yes | 0.0 | 0.0 | 0.0 | 0.0 | **yes** |  | yes | 0.0 | yes | 0.0 | yes | 0.0 | yes | 0.0 |
| Lysophosphatidylcholine (acyl) C24:0 | lysoPC a C24:0 | yes | 66.0 | 0.0 | 0.0 | 66.0 | **no** |  | yes | 0.0 | no | 100.0 | no | 100.0 | yes | 0.0 |
| Lysophosphatidylcholine (acyl) C26:0 | lysoPC a C26:0 | no | 100.0 | 0.0 | 0.0 | 100.0 | **no** |  | no | 100.0 | no | 100.0 | no | 100.0 | no | 100.0 |
| Lysophosphatidylcholine (acyl) C26:1 | lysoPC a C26:1 | no | 100.0 | 0.0 | 0.0 | 100.0 | **no** |  | no | 100.0 | no | 100.0 | no | 100.0 | no | 100.0 |
| Lysophosphatidylcholine (acyl) C28:0 | lysoPC a C28:0 | no | 100.0 | 0.0 | 0.0 | 100.0 | **no** |  | no | 100.0 | no | 100.0 | no | 100.0 | no | 100.0 |
| Lysophosphatidylcholine (acyl) C28:1 | lysoPC a C28:1 | yes | 0.0 | 0.0 | 0.0 | 0.0 | **yes** |  | yes | 0.0 | yes | 0.0 | yes | 0.0 | yes | 0.0 |
| Phosphatidylcholine (diacyl) C24:0 | PC aa C24:0 | no | 100.0 | 0.0 | 0.0 | 100.0 | **no** |  | no | 100.0 | no | 100.0 | no | 100.0 | no | 100.0 |
| Phosphatidylcholine (diacyl) C26:0 | PC aa C26:0 | no | 100.0 | 0.0 | 0.0 | 100.0 | **no** |  | no | 100.0 | no | 100.0 | no | 100.0 | no | 100.0 |
| Phosphatidylcholine (diacyl) C28:1 | PC aa C28:1 | yes | 0.0 | 0.0 | 0.0 | 0.0 | **yes** |  | yes | 0.0 | yes | 0.0 | yes | 0.0 | yes | 0.0 |
| Phosphatidylcholine (diacyl) C30:0 | PC aa C30:0 | yes | 0.0 | 0.0 | 0.0 | 0.0 | **yes** |  | yes | 0.0 | yes | 0.0 | yes | 0.0 | yes | 0.0 |
| Phosphatidylcholine (diacyl) C30:2 | PC aa C30:2 | no | 100.0 | 0.0 | 0.0 | 100.0 | **no** |  | no | 100.0 | no | 100.0 | no | 100.0 | no | 100.0 |
| Phosphatidylcholine (diacyl) C32:0 | PC aa C32:0 | yes | 0.0 | 0.0 | 0.0 | 0.0 | **yes** |  | yes | 0.0 | yes | 0.0 | yes | 0.0 | yes | 0.0 |
| Phosphatidylcholine (diacyl) C32:1 | PC aa C32:1 | yes | 0.0 | 0.0 | 0.0 | 0.0 | **yes** |  | yes | 0.0 | yes | 0.0 | yes | 0.0 | yes | 0.0 |
| Phosphatidylcholine (diacyl) C32:2 | PC aa C32:2 | yes | 0.0 | 0.0 | 0.0 | 0.0 | **yes** |  | yes | 0.0 | yes | 0.0 | yes | 0.0 | yes | 0.0 |
| Phosphatidylcholine (diacyl) C32:3 | PC aa C32:3 | yes | 0.0 | 0.0 | 0.0 | 0.0 | **yes** |  | yes | 0.0 | yes | 0.0 | yes | 0.0 | yes | 0.0 |
| Phosphatidylcholine (diacyl) C34:1 | PC aa C34:1 | yes | 0.0 | 0.0 | 0.0 | 0.0 | **yes** |  | yes | 0.0 | yes | 0.0 | yes | 0.0 | yes | 0.0 |
| Phosphatidylcholine (diacyl) C34:2 | PC aa C34:2 | yes | 0.0 | 0.0 | 0.0 | 0.0 | **yes** |  | yes | 0.0 | yes | 0.0 | yes | 0.0 | yes | 0.0 |
| Phosphatidylcholine (diacyl) C34:3 | PC aa C34:3 | yes | 0.0 | 0.0 | 0.0 | 0.0 | **yes** |  | yes | 0.0 | yes | 0.0 | yes | 0.0 | yes | 0.0 |
| Phosphatidylcholine (diacyl) C34:4 | PC aa C34:4 | yes | 0.0 | 0.0 | 0.0 | 0.0 | **yes** |  | yes | 0.0 | yes | 0.0 | yes | 0.0 | yes | 0.0 |
| Phosphatidylcholine (diacyl) C36:0 | PC aa C36:0 | yes | 0.0 | 0.0 | 0.0 | 0.0 | **yes** |  | yes | 0.0 | yes | 0.0 | yes | 0.0 | yes | 0.0 |
| Phosphatidylcholine (diacyl) C36:1 | PC aa C36:1 | yes | 0.0 | 0.0 | 0.0 | 0.0 | **yes** |  | yes | 0.0 | yes | 0.0 | yes | 0.0 | yes | 0.0 |
| Phosphatidylcholine (diacyl) C36:2 | PC aa C36:2 | yes | 0.0 | 0.0 | 0.0 | 0.0 | **yes** |  | yes | 0.0 | yes | 0.0 | yes | 0.0 | yes | 0.0 |
| Phosphatidylcholine (diacyl) C36:3 | PC aa C36:3 | yes | 0.0 | 0.0 | 0.0 | 0.0 | **yes** |  | yes | 0.0 | yes | 0.0 | yes | 0.0 | yes | 0.0 |
| Phosphatidylcholine (diacyl) C36:4 | PC aa C36:4 | yes | 0.0 | 0.0 | 0.0 | 0.0 | **yes** |  | yes | 0.0 | yes | 0.0 | yes | 0.0 | yes | 0.0 |
| Phosphatidylcholine (diacyl) C36:5 | PC aa C36:5 | yes | 0.0 | 0.0 | 0.0 | 0.0 | **yes** |  | yes | 0.0 | yes | 0.0 | yes | 0.0 | yes | 0.0 |
| Phosphatidylcholine (diacyl) C36:6 | PC aa C36:6 | yes | 0.0 | 0.0 | 0.0 | 0.0 | **yes** |  | yes | 0.0 | yes | 0.0 | yes | 0.0 | yes | 0.0 |
| Phosphatidylcholine (diacyl) C38:0 | PC aa C38:0 | yes | 0.0 | 0.0 | 0.0 | 0.0 | **yes** |  | yes | 0.0 | yes | 0.0 | yes | 0.0 | yes | 0.0 |
| Phosphatidylcholine (diacyl) C38:1 | PC aa C38:1 | no | 100.0 | 0.0 | 0.0 | 100.0 | **no** |  | no | 100.0 | no | 100.0 | no | 100.0 | no | 100.0 |
| Phosphatidylcholine (diacyl) C38:3 | PC aa C38:3 | yes | 0.0 | 0.0 | 0.0 | 0.0 | **yes** |  | yes | 0.0 | yes | 0.0 | yes | 0.0 | yes | 0.0 |
| Phosphatidylcholine (diacyl) C38:4 | PC aa C38:4 | yes | 0.0 | 0.0 | 0.0 | 0.0 | **yes** |  | yes | 0.0 | yes | 0.0 | yes | 0.0 | yes | 0.0 |
| Phosphatidylcholine (diacyl) C38:5 | PC aa C38:5 | yes | 0.0 | 0.0 | 0.0 | 0.0 | **yes** |  | yes | 0.0 | yes | 0.0 | yes | 0.0 | yes | 0.0 |
| Phosphatidylcholine (diacyl) C38:6 | PC aa C38:6 | yes | 0.0 | 0.0 | 0.0 | 0.0 | **yes** |  | yes | 0.0 | yes | 0.0 | yes | 0.0 | yes | 0.0 |
| Phosphatidylcholine (diacyl) C40:1 | PC aa C40:1 | yes | 0.0 | 76.9 | 0.0 | 76.9 | **no** |  | yes | 65.0 | yes | 72.8 | yes | 86.7 | yes | 83.9 |
| Phosphatidylcholine (diacyl) C40:2 | PC aa C40:2 | yes | 0.0 | 0.0 | 0.0 | 0.0 | **yes** |  | yes | 0.0 | yes | 0.0 | yes | 0.0 | yes | 0.0 |
| Phosphatidylcholine (diacyl) C40:3 | PC aa C40:3 | yes | 0.0 | 0.0 | 0.0 | 0.0 | **yes** |  | yes | 0.0 | yes | 0.0 | yes | 0.0 | yes | 0.0 |
| Phosphatidylcholine (diacyl) C40:4 | PC aa C40:4 | yes | 0.0 | 0.0 | 0.0 | 0.0 | **yes** |  | yes | 0.0 | yes | 0.0 | yes | 0.0 | yes | 0.0 |
| Phosphatidylcholine (diacyl) C40:5 | PC aa C40:5 | yes | 0.0 | 0.0 | 0.0 | 0.0 | **yes** |  | yes | 0.0 | yes | 0.0 | yes | 0.0 | yes | 0.0 |
| Phosphatidylcholine (diacyl) C40:6 | PC aa C40:6 | yes | 0.0 | 0.0 | 0.0 | 0.0 | **yes** |  | yes | 0.0 | yes | 0.0 | yes | 0.0 | yes | 0.0 |
| Phosphatidylcholine (diacyl) C42:0 | PC aa C42:0 | yes | 0.0 | 0.0 | 0.0 | 0.0 | **yes** |  | yes | 0.0 | yes | 0.0 | yes | 0.0 | yes | 0.0 |
| Phosphatidylcholine (diacyl) C42:1 | PC aa C42:1 | yes | 0.0 | 0.0 | 0.0 | 0.0 | **yes** |  | yes | 0.0 | yes | 0.0 | yes | 0.0 | yes | 0.0 |
| Phosphatidylcholine (diacyl) C42:2 | PC aa C42:2 | yes | 0.0 | 0.9 | 0.0 | 0.9 | **yes** |  | yes | 0.0 | yes | 0.0 | yes | 2.5 | yes | 0.0 |
| Phosphatidylcholine (diacyl) C42:4 | PC aa C42:4 | yes | 0.0 | 0.0 | 0.0 | 0.0 | **yes** |  | yes | 0.0 | yes | 0.0 | yes | 0.0 | yes | 0.0 |
| Phosphatidylcholine (diacyl) C42:5 | PC aa C42:5 | yes | 0.0 | 0.0 | 0.0 | 0.0 | **yes** |  | yes | 0.0 | yes | 0.0 | yes | 0.0 | yes | 0.0 |
| Phosphatidylcholine (diacyl) C42:6 | PC aa C42:6 | yes | 3.0 | 4.3 | 0.0 | 7.3 | **yes** |  | yes | 0.0 | yes | 2.9 | yes | 8.1 | yes | 5.4 |
| Phosphatidylcholine (acyl-alkyl) C30:0 | PC ae C30:0 | yes | 0.0 | 0.0 | 0.0 | 0.0 | **yes** |  | yes | 0.0 | yes | 0.0 | yes | 0.0 | yes | 0.0 |
| Phosphatidylcholine (acyl-alkyl) C30:1 | PC ae C30:1 | no | 100.0 | 0.0 | 0.0 | 100.0 | **no** |  | no | 100.0 | no | 100.0 | no | 100.0 | no | 100.0 |
| Phosphatidylcholine (acyl-alkyl) C30:2 | PC ae C30:2 | yes | 0.0 | 0.0 | 0.0 | 0.0 | **yes** |  | yes | 0.0 | yes | 0.0 | yes | 0.0 | yes | 0.0 |
| Phosphatidylcholine (acyl-alkyl) C32:1 | PC ae C32:1 | yes | 0.0 | 0.0 | 0.0 | 0.0 | **yes** |  | yes | 0.0 | yes | 0.0 | yes | 0.0 | yes | 0.0 |
| Phosphatidylcholine (acyl-alkyl) C32:2 | PC ae C32:2 | yes | 0.0 | 0.0 | 0.0 | 0.0 | **yes** |  | yes | 0.0 | yes | 0.0 | yes | 0.0 | yes | 0.0 |
| Phosphatidylcholine (acyl-alkyl) C34:0 | PC ae C34:0 | yes | 0.0 | 0.0 | 0.0 | 0.0 | **yes** |  | yes | 0.0 | yes | 0.0 | yes | 0.0 | yes | 0.0 |
| Phosphatidylcholine (acyl-alkyl) C34:1 | PC ae C34:1 | yes | 0.0 | 0.0 | 0.0 | 0.0 | **yes** |  | yes | 0.0 | yes | 0.0 | yes | 0.0 | yes | 0.0 |
| Phosphatidylcholine (acyl-alkyl) C34:2 | PC ae C34:2 | yes | 0.0 | 0.0 | 0.0 | 0.0 | **yes** |  | yes | 0.0 | yes | 0.0 | yes | 0.0 | yes | 0.0 |
| Phosphatidylcholine (acyl-alkyl) C34:3 | PC ae C34:3 | yes | 0.0 | 0.0 | 0.0 | 0.0 | **yes** |  | yes | 0.0 | yes | 0.0 | yes | 0.0 | yes | 0.0 |
| Phosphatidylcholine (acyl-alkyl) C36:0 | PC ae C36:0 | yes | 0.0 | 0.0 | 0.0 | 0.0 | **yes** |  | yes | 0.0 | yes | 0.0 | yes | 0.0 | yes | 0.0 |
| Phosphatidylcholine (acyl-alkyl) C36:1 | PC ae C36:1 | yes | 0.0 | 0.0 | 0.0 | 0.0 | **yes** |  | yes | 0.0 | yes | 0.0 | yes | 0.0 | yes | 0.0 |
| Phosphatidylcholine (acyl-alkyl) C36:2 | PC ae C36:2 | yes | 0.0 | 0.0 | 0.0 | 0.0 | **yes** |  | yes | 0.0 | yes | 0.0 | yes | 0.0 | yes | 0.0 |
| Phosphatidylcholine (acyl-alkyl) C36:3 | PC ae C36:3 | yes | 0.0 | 0.0 | 0.0 | 0.0 | **yes** |  | yes | 0.0 | yes | 0.0 | yes | 0.0 | yes | 0.0 |
| Phosphatidylcholine (acyl-alkyl) C36:4 | PC ae C36:4 | yes | 0.0 | 0.0 | 0.0 | 0.0 | **yes** |  | yes | 0.0 | yes | 0.0 | yes | 0.0 | yes | 0.0 |
| Phosphatidylcholine (acyl-alkyl) C36:5 | PC ae C36:5 | yes | 0.0 | 0.0 | 0.0 | 0.0 | **yes** |  | yes | 0.0 | yes | 0.0 | yes | 0.0 | yes | 0.0 |
| Phosphatidylcholine (acyl-alkyl) C38:0 | PC ae C38:0 | yes | 0.0 | 0.0 | 0.0 | 0.0 | **yes** |  | yes | 0.0 | yes | 0.0 | yes | 0.0 | yes | 0.0 |
| Phosphatidylcholine (acyl-alkyl) C38:1 | PC ae C38:1 | no | 100.0 | 0.0 | 0.0 | 100.0 | **no** |  | no | 100.0 | no | 100.0 | no | 100.0 | no | 100.0 |
| Phosphatidylcholine (acyl-alkyl) C38:2 | PC ae C38:2 | yes | 0.0 | 0.0 | 0.0 | 0.0 | **yes** |  | yes | 0.0 | yes | 0.0 | yes | 0.0 | yes | 0.0 |
| Phosphatidylcholine (acyl-alkyl) C38:3 | PC ae C38:3 | yes | 0.0 | 0.0 | 0.0 | 0.0 | **yes** |  | yes | 0.0 | yes | 0.0 | yes | 0.0 | yes | 0.0 |
| Phosphatidylcholine (acyl-alkyl) C38:4 | PC ae C38:4 | yes | 0.0 | 0.0 | 0.0 | 0.0 | **yes** |  | yes | 0.0 | yes | 0.0 | yes | 0.0 | yes | 0.0 |
| Phosphatidylcholine (acyl-alkyl) C38:5 | PC ae C38:5 | yes | 0.0 | 0.0 | 0.0 | 0.0 | **yes** |  | yes | 0.0 | yes | 0.0 | yes | 0.0 | yes | 0.0 |
| Phosphatidylcholine (acyl-alkyl) C38:6 | PC ae C38:6 | yes | 0.0 | 0.0 | 0.0 | 0.0 | **yes** |  | yes | 0.0 | yes | 0.0 | yes | 0.0 | yes | 0.0 |
| Phosphatidylcholine (acyl-alkyl) C40:1 | PC ae C40:1 | yes | 0.0 | 0.0 | 0.0 | 0.0 | **yes** |  | yes | 0.0 | yes | 0.0 | yes | 0.0 | yes | 0.0 |
| Phosphatidylcholine (acyl-alkyl) C40:2 | PC ae C40:2 | yes | 0.0 | 0.0 | 0.0 | 0.0 | **yes** |  | yes | 0.0 | yes | 0.0 | yes | 0.0 | yes | 0.0 |
| Phosphatidylcholine (acyl-alkyl) C40:3 | PC ae C40:3 | yes | 0.0 | 0.0 | 0.0 | 0.0 | **yes** |  | yes | 0.0 | yes | 0.0 | yes | 0.0 | yes | 0.0 |
| Phosphatidylcholine (acyl-alkyl) C40:4 | PC ae C40:4 | no | 100.0 | 0.0 | 0.0 | 100.0 | **no** |  | no | 100.0 | no | 100.0 | no | 100.0 | no | 100.0 |
| Phosphatidylcholine (acyl-alkyl) C40:5 | PC ae C40:5 | yes | 0.0 | 0.0 | 0.0 | 0.0 | **yes** |  | yes | 0.0 | yes | 0.0 | yes | 0.0 | yes | 0.0 |
| Phosphatidylcholine (acyl-alkyl) C40:6 | PC ae C40:6 | yes | 0.0 | 0.0 | 0.0 | 0.0 | **yes** |  | yes | 0.0 | yes | 0.0 | yes | 0.0 | yes | 0.0 |
| Phosphatidylcholine (acyl-alkyl) C42:0 | PC ae C42:0 | yes | 0.0 | 0.0 | 0.0 | 0.0 | **yes** |  | yes | 0.0 | yes | 0.0 | yes | 0.0 | yes | 0.0 |
| Phosphatidylcholine (acyl-alkyl) C42:1 | PC ae C42:1 | yes | 0.0 | 0.9 | 0.0 | 0.9 | **yes** |  | yes | 0.0 | yes | 0.0 | yes | 2.5 | yes | 0.0 |
| Phosphatidylcholine (acyl-alkyl) C42:2 | PC ae C42:2 | yes | 0.0 | 0.0 | 0.0 | 0.0 | **yes** |  | yes | 0.0 | yes | 0.0 | yes | 0.0 | yes | 0.0 |
| Phosphatidylcholine (acyl-alkyl) C42:3 | PC ae C42:3 | yes | 0.0 | 0.0 | 0.0 | 0.0 | **yes** |  | yes | 0.0 | yes | 0.0 | yes | 0.0 | yes | 0.0 |
| Phosphatidylcholine (acyl-alkyl) C42:4 | PC ae C42:4 | yes | 0.0 | 0.0 | 0.0 | 0.0 | **yes** |  | yes | 0.0 | yes | 0.0 | yes | 0.0 | yes | 0.0 |
| Phosphatidylcholine (acyl-alkyl) C42:5 | PC ae C42:5 | yes | 0.0 | 0.0 | 0.0 | 0.0 | **yes** |  | yes | 0.0 | yes | 0.0 | yes | 0.0 | yes | 0.0 |
| Phosphatidylcholine (acyl-alkyl) C44:3 | PC ae C44:3 | yes | 2.3 | 0.5 | 0.0 | 2.8 | **yes** |  | yes | 0.0 | yes | 1.3 | yes | 0.7 | yes | 0.0 |
| Phosphatidylcholine (acyl-alkyl) C44:4 | PC ae C44:4 | yes | 0.0 | 0.1 | 0.0 | 0.1 | **yes** |  | yes | 0.0 | yes | 0.0 | yes | 0.4 | yes | 0.0 |
| Phosphatidylcholine (acyl-alkyl) C44:5 | PC ae C44:5 | yes | 0.0 | 0.0 | 0.0 | 0.0 | **yes** |  | yes | 0.0 | yes | 0.0 | yes | 0.0 | yes | 0.0 |
| Phosphatidylcholine (acyl-alkyl) C44:6 | PC ae C44:6 | yes | 0.0 | 0.0 | 0.0 | 0.0 | **yes** |  | yes | 0.0 | yes | 0.0 | yes | 0.0 | yes | 0.0 |
| **SPHINGOLIPIDS** |  |  |  |  |  |  |  |  |  |  |  |  |  |  |  |  |
| Hydroxysphingomyelin C14:1 | SM (OH) C14:1 | yes | 0.0 | 0.0 | 0.0 | 0.0 | **yes** |  | yes | 0.0 | yes | 0.0 | yes | 0.0 | yes | 0.0 |
| Hydroxysphingomyelin C16:1 | SM (OH) C16:1 | yes | 0.0 | 0.0 | 0.0 | 0.0 | **yes** |  | yes | 0.0 | yes | 0.0 | yes | 0.0 | yes | 0.0 |
| Hydroxysphingomyelin C22:1 | SM (OH) C22:1 | yes | 0.0 | 0.0 | 0.0 | 0.0 | **yes** |  | yes | 0.0 | yes | 0.0 | yes | 0.0 | yes | 0.0 |
| Hydroxysphingomyelin C22:2 | SM (OH) C22:2 | yes | 0.0 | 0.0 | 0.0 | 0.0 | **yes** |  | yes | 0.0 | yes | 0.0 | yes | 0.0 | yes | 0.0 |
| Hydroxysphingomyelin C24:1 | SM (OH) C24:1 | yes | 0.0 | 0.0 | 0.0 | 0.0 | **yes** |  | yes | 0.0 | yes | 0.0 | yes | 0.0 | yes | 0.0 |
| Sphingomyelin C16:0 | SM C16:0 | yes | 0.0 | 0.0 | 0.0 | 0.0 | **yes** |  | yes | 0.0 | yes | 0.0 | yes | 0.0 | yes | 0.0 |
| Sphingomyelin C16:1 | SM C16:1 | yes | 0.0 | 0.0 | 0.0 | 0.0 | **yes** |  | yes | 0.0 | yes | 0.0 | yes | 0.0 | yes | 0.0 |
| Sphingomyelin C18:0 | SM C18:0 | yes | 0.0 | 0.0 | 0.0 | 0.0 | **yes** |  | yes | 0.0 | yes | 0.0 | yes | 0.0 | yes | 0.0 |
| Sphingomyelin C18:1 | SM C18:1 | yes | 0.0 | 0.0 | 0.0 | 0.0 | **yes** |  | yes | 0.0 | yes | 0.0 | yes | 0.0 | yes | 0.0 |
| Sphingomyelin C20:2 | SM C20:2 | yes | 0.0 | 0.3 | 0.0 | 0.3 | **yes** |  | yes | 0.0 | yes | 0.0 | yes | 0.7 | yes | 0.0 |
| Sphingomyelin C22:3 | SM C22:3 | no | 100.0 | 0.0 | 0.0 | 100.0 | **no** |  | no | 100.0 | no | 100.0 | no | 100.0 | no | 100.0 |
| Sphingomyelin C24:0 | SM C24:0 | yes | 0.0 | 0.0 | 0.0 | 0.0 | **yes** |  | yes | 0.0 | yes | 0.0 | yes | 0.0 | yes | 0.0 |
| Sphingomyelin C24:1 | SM C24:1 | yes | 0.0 | 0.0 | 0.0 | 0.0 | **yes** |  | yes | 0.0 | yes | 0.0 | yes | 0.0 | yes | 0.0 |
| Sphingomyelin C26:0 | SM C26:0 | yes | 0.0 | 0.0 | 0.0 | 0.0 | **yes** |  | yes | 0.0 | yes | 0.0 | yes | 0.0 | yes | 0.0 |
| Sphingomyelin C26:1 | SM C26:1 | yes | 0.0 | 0.0 | 0.0 | 0.0 | **yes** |  | yes | 0.0 | yes | 0.0 | yes | 0.0 | yes | 0.0 |
| **HEXOSES** |  |  |  |  |  |  |  |  |  |  |  |  |  |  |  |  |
| Hexoses | H1 | yes | 0.0 | 0.9 | 0.0 | 0.9 | **yes** |  | yes | 0.0 | yes | 0.0 | yes | 2.1 | yes | 1.8 |
| TOTAL NUMBER | 186 |  |  |  |  |  | **130** |  | 147 |  | 148 |  | 146 |  | 149 |  |

*LOD/LOQ: Level of detection/Level of quantification, LOD values are used for acylcarnitines, glycerophospho- and sphingolipids while LOQ values are used for amino acids, biogenic amines and sugar; **ULOD: Upper level of detection.
