# Supplementary material for: Plasma metabolites associated with colorectal cancer stage: Findings from an international consortium
Source: Int J Cancer. 2019 Oct 10;146(12):3256–66. doi: 10.1002/ijc.32666 (PMC7216900; doi:10.1002/ijc.32666)
Supplement: Supplementary file 2 — Supplementary Table S2 Baseline clinicodemographic and lifestyle characteristics of the total study population and by individual cohort [file IJC-146-3256-s002.docx]

**Supplementary Table S2.** Baseline clinicodemographic and lifestyle characteristics of the total study population and by individual cohort

|  |  | *Cohorts* | | | | | | | |  | *Total population  (n= 744)* | |
| --- | --- | --- | --- | --- | --- | --- | --- | --- | --- | --- | --- | --- |
|  |  | **COLON** (n= 197) | | **EnCoRe** (n= 206) | | **ColoCare** (n= 285) | | **CORSA** (n= 56) | |  |  |  |
| Male sex, n(%) |  | 116 | (58.9) | 134 | (65.0) | 190 | (66.7) | 43 | (76.8) |  | **483** | **(64.9)** |
| Age^*^, median (IQR) |  | 67.0 | (62.0 – 72.0) | 67.0 | (60.0 – 73.8) | 64.0 | (55.0 – 72.0) | 68.0 | (58.5 – 75.3) |  | **66.0** | **(59.0 – 73.0)** |
| Body mass index^*^, kg/m^2^ |  |  |  |  |  |  |  |  |  |  |  |  |
| Median^*^ (IQR) |  | 25.5 | (23.2 – 28.0) | 27.9 | (25.6 – 30.8) | 26.1 | (23.7 – 28.7) | 26.2 | (23.9 – 29.5) |  | **26.5** | **(24.0 – 29.4)** |
| Underweight, <18.5 n(%) |  | 3 | (1.5) | 1 | (0.5) | 6 | (2.1) | 0 | (0.0) |  | **10** | **(1.3)** |
| Normal weight, 18.5-24.9 n(%) |  | 85 | (43.1) | 44 | (21.4) | 98 | (34.4) | 18 | (32.1) |  | **245** | **(32.9)** |
| Overweight, 25-29.9 n(%) |  | 81 | (41.1) | 94 | (45.6) | 128 | (44.9) | 27 | (48.2) |  | **330** | **(44.4)** |
| Obese, ≥30 n(%) |  | 28 | (14.2) | 67 | (32.5) | 53 | (18.6) | 11 | (19.6) |  | **159** | **(21.4)** |
| Smoking status^a,*^, n(%) |  |  |  |  |  |  |  |  |  |  |  |  |
| Current |  | 19 | (9.6) | 27 | (13.1) | 52 | (18.2) | 13 | (23.2) |  | **111** | **(14.9)** |
| Former |  | 119 | (60.4) | 110 | (53.4) | 121 | (42.5) | 19 | (33.9) |  | **369** | **(49.6)** |
| Never |  | 58 | (29.4) | 64 | (31.1) | 99 | (34.7) | 23 | (41.1) |  | **244** | **(32.8)** |
| Stage of disease^b,*^, n(%) |  |  |  |  |  |  |  |  |  |  |  |  |
| I |  | 53 | (26.9) | 54 | (26.2) | 41 | (14.4) | 20 | (35.7) |  | **168** | **(22.6)** |
| II |  | 66 | (33.5) | 50 | (24.3) | 83 | (29.1) | 13 | (23.2) |  | **212** | **(28.5)** |
| III |  | 71 | (36.0) | 102 | (49.5) | 103 | (36.1) | 14 | (25.0) |  | **290** | **(39.0)** |
| IV |  | 7 | (3.6) | 0 | (0.0) | 58 | (20.4) | 9 | (16.1) |  | **74** | **(9.9)** |
| Tumour site^c,d,*^, n(%) |  |  |  |  |  |  |  |  |  |  |  |  |
| Colon – proximal |  | 57 | (28.9) | 57 | (27.7) | 71 | (24.9) | 20 | (35.7) |  | **205** | **(27.6)** |
| Colon – distal |  | 71 | (36.0) | 70 | (34.0) | 65 | (22.8) | 18 | (32.1) |  | **224** | **(30.1)** |
| Rectal |  | 69 | (35.0) | 79 | (38.3) | 149 | (52.3) | 16 | (28.6) |  | **313** | **(42.1)** |
| Treatment, n(%) |  |  |  |  |  |  |  |  |  |  |  |  |
| Neo-adjuvant therapy^*^ |  | 61 | (31.0) | 60 | (29.1) | 86 | (30.2) | 1 | (1.8) |  | **208** | **(28.0)** |
| Surgery^*^ |  | 197 | (100.0) | 196 | (95.1) | 285 | (100.0) | 54 | (96.4) |  | **732** | **(98.4)** |

^a^ Missing data for 5, 13 and 1 patients of the EnCoRe, ColoCare and CORSA study, respectively;  ^b^ pTNM for patients who underwent surgery, cTNM for patients with rectal cancer who received neo-adjuvant therapy and patients with colon cancer without surgery; ^c^ Proximal consisting of: hepatic flexure, transverse colon, cecum, appendix, ascending colon; Distal consisting of: descending colon, sigmoid colon, splenic flexure; Rectal consisting of: rectosigmoid junction, rectum; ^d^ Missing data for 2 patients of the CORSA study.
*Statistically significant differences between cohorts (*p*<0.05) using Kruskal-Wallis tests or Chi-Square Tests.
