# Supplementary material for: Plasma metabolites associated with colorectal cancer stage: Findings from an international consortium
Source: Int J Cancer. 2019 Oct 10;146(12):3256–66. doi: 10.1002/ijc.32666 (PMC7216900; doi:10.1002/ijc.32666)
Supplement: Supplementary file 7 — Supplementary Figure S1 Weighted and overall Rpartial 2 for covariates showing the percentage of variability explained by each covariate separately and combined in metabolite concentrations, from the PC‐PR2 analysis. Included covariates were stage (I/II/III/IV), smoking status (current/former/never), tumor site (distal/proximal/rectal), analytical batch (1‐19), sex, cohort (COLON/EnCoRe/ColoCare/ CORSA), age and body mass index (continuous). [file IJC-146-3256-s007.docx]

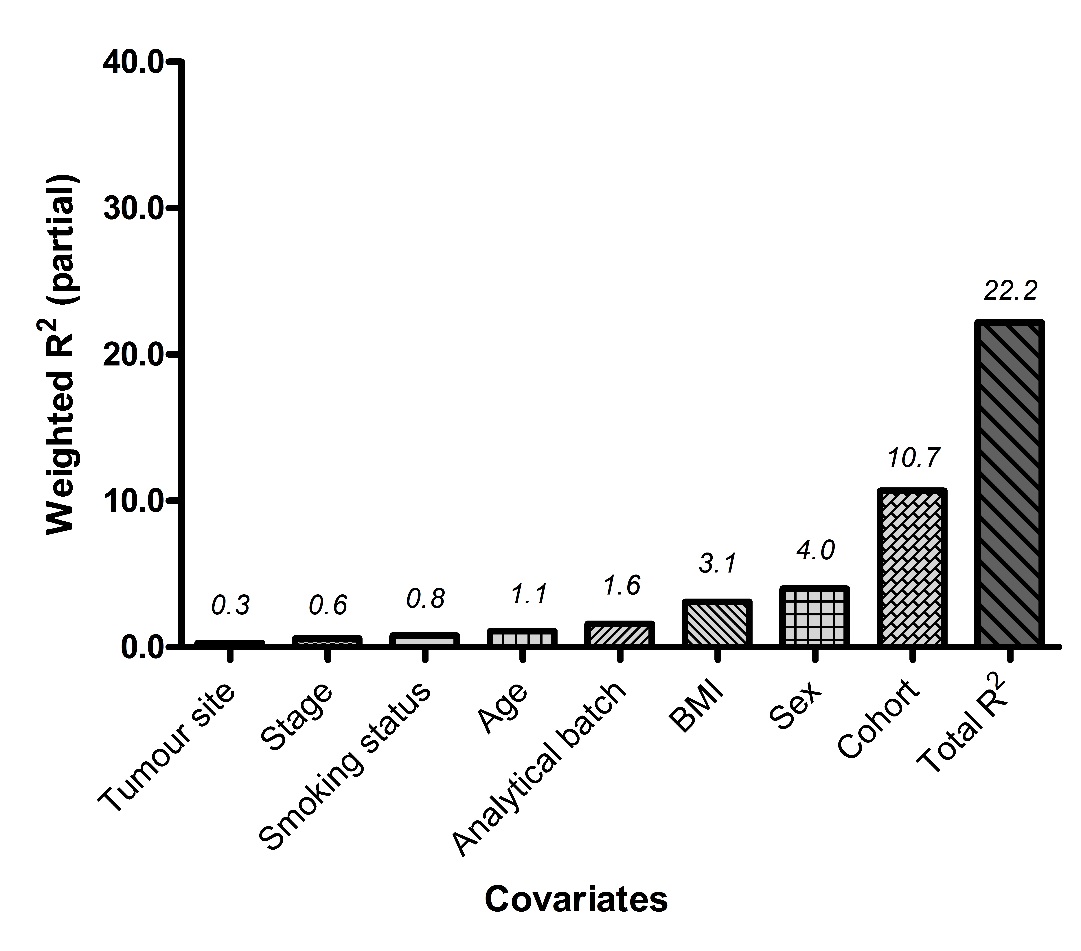


**Supplementary Figure S1**. Weighted and overall R_partial_^2^ for covariates showing the percentage of variability explained by each covariate separately and combined in metabolite concentrations, from the PC-PR2 analysis. Included covariates were stage (I/II/III/IV), smoking status (current/former/never), tumour site (distal/proximal/rectal), analytical batch (1-19), sex, cohort (COLON/EnCoRe/ColoCare/ CORSA), age and body mass index (continuous).
